# A Network-Based Analysis Reveals the Mechanism Underlying Vitamin D in Suppressing Cytokine Storm and Virus in SARS-CoV-2 Infection

**DOI:** 10.3389/fimmu.2020.590459

**Published:** 2020-12-09

**Authors:** Firoz Ahmed

**Affiliations:** ^1^ Department of Biochemistry, College of Science, University of Jeddah, Jeddah, Saudi Arabia; ^2^ University of Jeddah Center for Scientific and Medical Research, University of Jeddah, Jeddah, Saudi Arabia

**Keywords:** SARS-CoV-2, COVID-19, cytokine storm, vitamin D, lung fibrosis, bioinformatics, regulatory network, RNA sequencing

## Abstract

**Background:**

SARS-CoV-2 causes ongoing pandemic coronavirus disease of 2019 (COVID-19), infects the cells of the lower respiratory tract that leads to a cytokine storm in a significant number of patients resulting in severe pneumonia, shortness of breathing, respiratory and organ failure. Extensive studies suggested the role of Vitamin D in suppressing cytokine storm in COVID-19 and reducing viral infection; however, the precise molecular mechanism is not clearly known. In this work, bioinformatics and systems biology approaches were used to understand SARS-CoV-2 induced cytokine pathways and the potential mechanism of Vitamin D in suppressing cytokine storm and enhancing antiviral response.

**Results:**

This study used transcriptome data and identified 108 differentially expressed host genes (DEHGs) in SARS-CoV-2 infected normal human bronchial epithelial (NHBE) cells compared to control. Then, the DEHGs was integrated with the human protein-protein interaction data to generate a SARS-CoV-2 induced host gene regulatory network (*SiHgrn*). Analysis of *SiHgrn* identified a sub-network “*Cluster 1*” with the highest MCODE score, 31 up-regulated genes, and predominantly associated immune and inflammatory response. Interestingly, the iRegulone tool identified that “*Cluster 1*” is under the regulation of transcription factors STAT1, STAT2, STAT3, POU2F2, and NFkB1, collectively referred to as “*host response signature network*”. Functional enrichment analysis with NDEx revealed that the “*host response signature network*” is predominantly associated with critical pathways, including “cytokines and inflammatory response”, “non-genomic action of Vitamin D”, “the human immune response to tuberculosis”, and “lung fibrosis”. Finally, in-depth analysis and literature mining revealed that Vitamin D binds with its receptor and could work through two different pathways: (i) it inhibits the expression of pro-inflammatory cytokines through blocking the TNF induced NFkB1 signaling pathway; and (ii) it initiates the expression of interferon-stimulating genes (ISGs) for antiviral defense program through activating the IFN-α induced Jak-STAT signaling pathway.

**Conclusion:**

This comprehensive study identified the pathways associated with cytokine storm in SARS-CoV-2 infection. The proposed underlying mechanism of Vitamin D could be promising in suppressing the cytokine storm and inducing a robust antiviral response in severe COVID-19 patients. The finding in this study urgently needs further experimental validations for the suitability of Vitamin D in combination with IFN-α to control severe COVID-19.

## Introduction

The emergence and rapid spread of a highly pathogenic new coronavirus, severe acute respiratory syndrome coronavirus 2 (SARS-CoV-2), created tremendous health, economic, and social crises worldwide. SARS-CoV-2 infects the cells of the lower respiratory tract and causes severe respiratory disease in humans, named coronavirus disease of 2019 (COVID-19). In late 2019, SARS-CoV-2 was reported in patients with severe pneumonia in Wuhan city of Hubei province, China. In a short period of time, the virus has infected a large number of people around the world, and this number is increasing exponentially (1). On March 11, 2020, the COVID-19 outbreak was declared a new pandemic by the World Health Organization (WHO). As of October 5, 2020, the number of SARS-CoV-2 infected people were 35,645,015; and among them, 1,044,898 cases of death and 26,791,820 cases of recovered were reported (https://www.worldometers.info/coronavirus/).

SARS-CoV-2 is closely related to previously identified pathogenic beta-coronavirus SARS-CoV, responsible for the epidemic in 2002 to 2003; and Middle East respiratory syndrome coronavirus (MERS-CoV), accountable for the epidemic in 2012 (1–4). The SARS-CoV-2 genome is about 29,000 nucleotides long positive-sense single-stranded RNA that encodes four structural proteins, sixteen non-structural proteins, and nine accessory proteins (1). These proteins help the virus to infect the host cell and hijack the host’s cellular machinery for virus assembly, amplification, and pathogenesis. During infection, the spike (S) protein of SARS-CoV-2 binds with host receptor angiotensin-converting enzyme 2 (ACE2) (5). Subsequently, host serine protease TMPRSS2 cleaves the viral S protein to generate S1 and S2 subunits, which fuses the viral and host membrane resulting internalization of the virus (5).

SARS-CoV-2–infected patients showed a symptom of fever, cough, and dyspnea. However, severe COVID-19 patients with comorbidities of pneumonia lead to acute respiratory distress syndrome (ARDS), and multi-organ failure resulting in death (1, 6–9). After SARS-CoV-2 infection, the lung cells activate the innate immune response against viruses. The severe COVID-19 cases show an influx of pro-inflammatory cytokines in the surrounding environment resulting in recruitments and stimulation of immune cells, including macrophages, neutrophils, and natural killer (NK) cells to kill virus-infected cells. The activated immune system regulation is important to target only virus-infected cells without harming healthy cells. Clinical data suggest that most COVID-19 deaths are due to the influx of various pro-inflammatory cytokines, termed as cytokine storm or cytokine release syndrome (CRS), which are responsible for severe damage of the healthy cells, tissues, and organs (6, 10). A study found an increase in Th17 cell and inflammatory cytokines (IL2, IL6, IL10, and IFN-γ) in severe COVID-19 patients compared to mild cases (10, 11). In contrast, severe COVID-19 cases reported a decrease in CD4^+^ cell, CD8^+^ cell, and lymphocytes compared to mild cases.

Previous studies identified that only 20% of COVID-19 patients showed the severe symptom of cytokine storm. Several studies indicate the role of Vitamin D supplements in protecting against diseases and reducing the risk of bacterial and viral infections, including influenza and SARS-CoV-2 infections (12–15). Vitamin D is a fat-soluble vitamin that involves maintaining the calcium level in the body. Classically, Vitamin D interacts with the nuclear Vitamin D receptor (VDR) to form a complex, which binds to the promoter region and modulates the expression of target genes (16). Besides, Vitamin D could also work through non-genomic action where it binds to the VDR and activates several signaling pathways, and indirectly regulates the transcription of numerous genes, including genes associated with immune response (12, 16). Numerous studies indicated that vitamin D deficiency is associated with cytokine storm and causes high morbidity and mortality in COVID-19 patients (14, 17). Moreover, the studies suggested that Vitamin D supplementation reduces the risk of the cytokine storm. However, the underlying molecular mechanism of Vitamin D in suppressing the cytokine storm and reducing viral infection in COVID-19 is not clearly understood yet, which is crucial to develop better therapy and management to save the life of SARS-CoV-2 infected person.

With the advancement of high-throughput “*omics*” technology and computational methods, the gene expression and protein interaction data could be integrated at the systems biology level to identify the altered gene regulatory network, activated pathways, and the molecular mechanism underlying complex genetic and infectious diseases (18–26).

In this work, bioinformatics and systems biology approaches were used to understand SARS-CoV-2 induced altered host gene regulatory network, biological pathways of cytokine storm, and the potential regulatory mechanism of Vitamin D in suppressing cytokine storm and reducing viral infection. The study used publicly available transcriptome data to identify the differentially expressed host genes (DEHGs) in SARS-CoV-2–infected human cells compared to mock-infected cells (control). Then, the biological role of DEHGs was analyzed with Gene Ontology (GO) and Kyoto Encyclopedia of Genes and Genomes (KEGG) pathways. Moreover, DEHGs were integrated with the human protein-protein interaction data (PPI). A gene regulatory network was created and analyzed to understand altered biological processes and their upstream master regulators. Our comprehensive analysis revealed a vital sub-network of highly interconnected 31 proteins predominantly responsible for the immune and inflammatory response and is under the regulation of transcription factors (TFs) STAT1, STAT2, STAT3, POU2F2, and NFkB1, which collectively referred to as “*host response signature network*”.

Furthermore, the “*host response signature network*” is significantly associated with altered biological pathways, including “cytokines and inflammatory response”, and “non-genomic action of Vitamin D”. Interestingly, our study revealed a potential mechanism through which Vitamin D could suppress cytokine storm and reduce viral infection in COVID-19. Finally, differentially expressed host genes from COVID-19 patients (DEHGs^COVID-19^) were taken to validate our findings. This study will contribute to a better understanding of the regulatory mechanism of cytokine storm and therapeutic intervention for severe COVID-19 patients.

## Materials and Methods

### Transcriptome Data and Identification of DEHGs

This study used the RNA sequencing data of normal human bronchial epithelial (NHBE) cells infected with SARS-CoV-2 (USA-WA1/2020) and mock-infected NHBE cells as control. The transcriptome data were downloaded from the Gene Expression Omnibus database (www.ncbi.nlm.nih.gov/geo/) with accession number GSE147507 (27). Each infected and controlled group has independent biological triplicate experimental data, and the reads were mapped on the human genome (hg19). The raw counts of expression data were used to identify the DEHGs between virus-infected cells compared to control with DESeq2 package version 1.22.2 in R version 3.5.2 (28). The DESeq2 takes two files as input: (a) a table of raw read counts from different samples where each row represents a gene and a column indicates the sample, and (b) an associated phenodata file describing the experimental group of each sample. DESeq2 performs internal normalization to adjust the differences in library size and library composition. For this, DESeq2 first calculates the scaling factor for each sample. Then the original read count is divided with sample scaling factor to get a normalized value. For each gene, DESeq2 uses a negative binomial distribution to model the counts and fit the normalized count data. DESeq2 uses shrinkage estimation for dispersions and fold changes and uses the Wald test to find out genes with significant differential expression between two sample groups.

**Figure 1:**
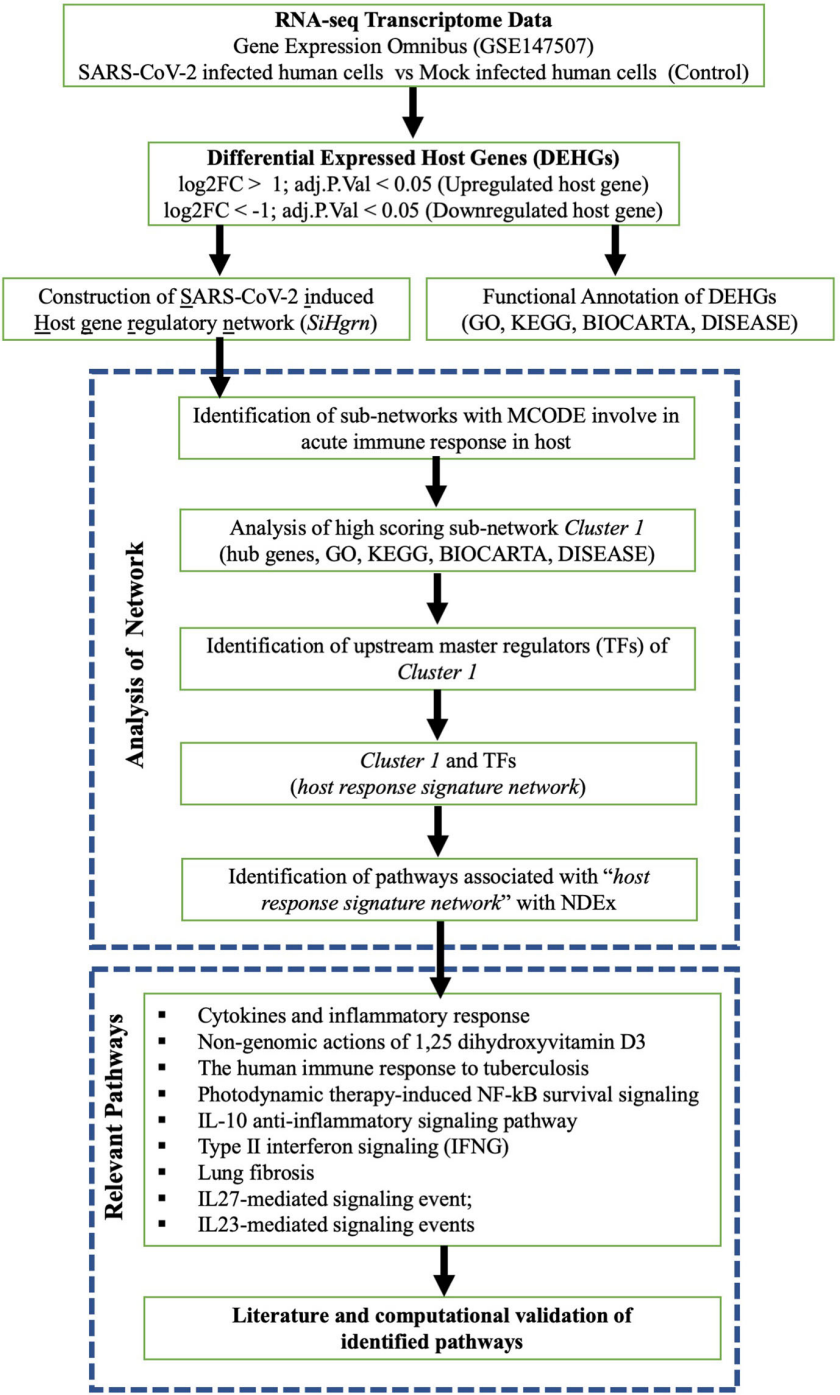
Schematic diagram of present study workflow.

A schematic diagram of our study is presented in **Figure 1**. The host gene is considered as differentially expressed if the log2 Fold Change |log2FC| is > 1 and adjusted p-value is < 0.05. The expression data of the significant DEHGs we selected and transformed into Z-score (row-wise of value), then a heatmap was created with pheatmap package version 1.0.12 in R.

### Functional Enrichment of DEHGs

To understand the biological processes induced by SARS-CoV-2, the list of DEHGs were analyzed for functional enrichment, including GO of Biological Process (BP), Molecular Function (MF), and Cellular Component (CC); KEGG and BIOCARTA pathways; and DISEASE using DAVID 6.8 (https://david.ncifcrf.gov/home.jsp). The significantly enriched terms were considered when at least five genes were present, and the Benjamini adjusted p-value is less than 0.05 (p-adjust <0.05). Then the enrichment was visualized using dotplot with the ggplot2 in R.

### Construction and Analysis of SARS-CoV-2–induced Host Gene Regulatory Network (SiHgrn)

The list of DEHGs, along with two additional host genes (ACE2 and TMPRSS2 involved in SARS-CoV-2 infection), was mapped to the STRING version 11 application (29). Although *ACE2* and *TMPRSS2* were not in the list of DEHGs, these two host proteins are nevertheless crucial for SARS-CoV-2 infection. Therefore, ACE2 and TMPRSS2 were taken into account during network construction to determine whether these proteins could interact with other proteins of DEHGs and influence the regulatory network. The human PPI data with medium confidence interaction score >0.400 was downloaded from STRING, and then a network was constructed and visualized in Cytoscape software version 3.7. In this network, each protein is represented by a node, while the interaction between two proteins is represented by an edge (30). Furthermore, the gene expression level (log2FC score) from DEHGs was integrated into the protein node to construct a PPI network called *SiHgrn*. After that, the topological structure of the *SiHgrn* was analyzed using Cytoscape plug-in “NetworkAnalyzer.”

The highly connected local sub-networks modules in the *SiHgrn* were extracted using the Cytoscape plug-in MCODE clustering algorithm (31). The functional enrichment of the highest score MCODE (*Cluster 1*) was analyzed for GO, KEGG, and BIOCARTA pathways, and DISEASE with DAVID 6.8. Besides, the gene set of *Cluster 1* was analyzed with Molecular Signatures Database (MSigDB version 7.1) (https://www.gsea-msigdb.org/gsea/msigdb/index.jsp), which is the most famous repository of biological gene sets for performing enrichment analysis (32). Each gene set contains a collection of genes that shared a specific-biological property. In this study, MSigBD was used to identify the common underlying biological process and pathways with “hallmark gene sets” and the “positional gene set” at default parameters. The “hallmark gene sets” is the collection of 50 gene sets in which each set contains a well-defined biological state or process with coordinate expression (33). The “positional gene sets” is the collection of 299 gene sets by chromosome and cytogenetic band, and thus, it can help identify effects related to the chromosomal position. Furthermore, MSigDB was also used to determine the gene family according to their protein homology or biochemical activity.

### Discovery of Upstream Master Regulators of Cluster 1

To find the upstream TFs regulators of genes of *Cluster 1*, Cytoscape plug-in iRegulone (version 1.3) was used at default parameters (34). The output result was further analyzed using the in house Perl program, and a matrix table was generated where each row indicates a TF, while a column indicates a target gene. The matrix was then visualized with a heatmap using the Morpheus tool (https://software.broadinstitute.org/morpheus/). The proteins of *Cluster 1* and upstream TFs are considered as a “*host response signature network*”.

### Biological Pathways of *“Host Response Signature Network”*

The gene set of “*host response signature network* (*Cluster 1* and its upstream regulators)” were analyzed to identify the biological pathways using the “Relevant Pathways” module of Network Data Exchange (NDEx version 2.4.5, 18 June 2020) (https://ndexbio.org/) (35, 36). NDEx is an open-source resource for the collection and analysis of biological network knowledge. The output result of “Relevant Pathways” analysis gave a list of biological pathways enriched for query genes. The result was sorted according to the highest similarity score between our query set and the network’s genes. Then, the top nine significantly enriched pathways having more than 5 overlapping genes were collected for further analysis. If required, figures from WikiPathways were modified with PathVisio version 3.3.0.

## Results

### Identification of DEHGs

In order to understand how the host gene expression altered in response to SARS-CoV-2 infection, the raw read counts of host transcriptome data were preprocessed and analyzed to identify the DEHGs in SARS-CoV-2–infected cell compared to control cell with DESeq2. The boxplots showed the raw read counts, and normalized read counts across samples (**Figure S1**). A global view of differentially expressed genes in RNA-seq datasets was visualized with the MA-plot, where M indicates the log_2_ fold change on the y-axis, while A indicates the mean of the normalized counts on the x-axis (**Figure S2**). A total of 108 DEHGs including 93 up-regulated and 15 down-regulated genes were identified with |log2FC| > 1 and adjusted p-value < 0.05. Among them, the top 15 genes showing up-regulation are *CSF3, SPRR2E*, *CXCL5*, *CCL20*, *SPRR2D*, *CSF2*, *IL6*, *IFI27*, *IL36G*, *XAF1*, *MX1*, *PDZK1IP1*, *SAA2*, *IFI6, and CXCL8*; while 15 genes showing down-regulation are *RBM20*, *IFITM10*, *NANOS1*, *STON1*, *CXCL14*, *MYLK*, *MAP7D2*, *NID1*, *ZNF488*, *ATG9B*, *VTCN1*, *CYP4F3*, *MIR221*, *PPARGC1A*, and *METTL7A* (**Table 1**). The detailed information of up- and down-regulated genes is provided in **Tables S1** and **S2**, respectively. Furthermore, a heatmap of gene expression level was generated, which shows a distinct pattern of up-regulated and down-regulated genes in SARS-CoV-2–infected cells compared with mock across all biological samples (**Figure S3**).

**Table 1:** A list of the top 15 differentially expressed host genes (DEHGs) in SARS-CoV-2–infected NHBE cells.

Up-regulated			Down-regulated		
Gene	Log2FC	Adj. p-value	Gene	Log2FC	Adj. p-value
*CSF3*	4.85	3.65E-20	*RBM20*	−1.58	3.93E-02
*SPRR2E*	3.60	4.59E-14	*IFITM10*	−1.57	3.39E-10
*CXCL5*	3.49	3.78E-28	*NANOS1*	−1.52	6.21E-03
*CCL20*	3.15	1.38E-76	*STON1*	−1.51	8.89E-03
*SPRR2D*	2.98	2.14E-48	*CXCL14*	−1.45	7.56E-06
*CSF2*	2.98	9.98E-11	*MYLK*	−1.43	7.58E-06
*IL6*	2.93	7.71E-24	*MAP7D2*	−1.39	6.13E-04
*IFI27*	2.90	2.18E-12	*NID1*	−1.23	6.75E-04
*IL36G*	2.73	3.86E-50	*ZNF488*	−1.14	1.54E-02
*XAF1*	2.53	4.03E-13	*ATG9B*	−1.13	3.40E-03
*MX1*	2.51	6.73E-34	*VTCN1*	−1.07	7.25E-06
*PDZK1IP1*	2.43	8.68E-21	*CYP4F3*	−1.07	1.65E-02
*SAA2*	2.42	9.38E-76	*MIR221*	−1.05	4.95E-02
*IFI6*	2.35	1.94E-03	*PPARGC1A*	−1.05	2.17E-03
*CXCL8(IL8)*	2.34	5.24E-105	*METTL7A*	−1.03	1.57E-05

### Functional Enrichment of DEHGs

**Figure 2:**
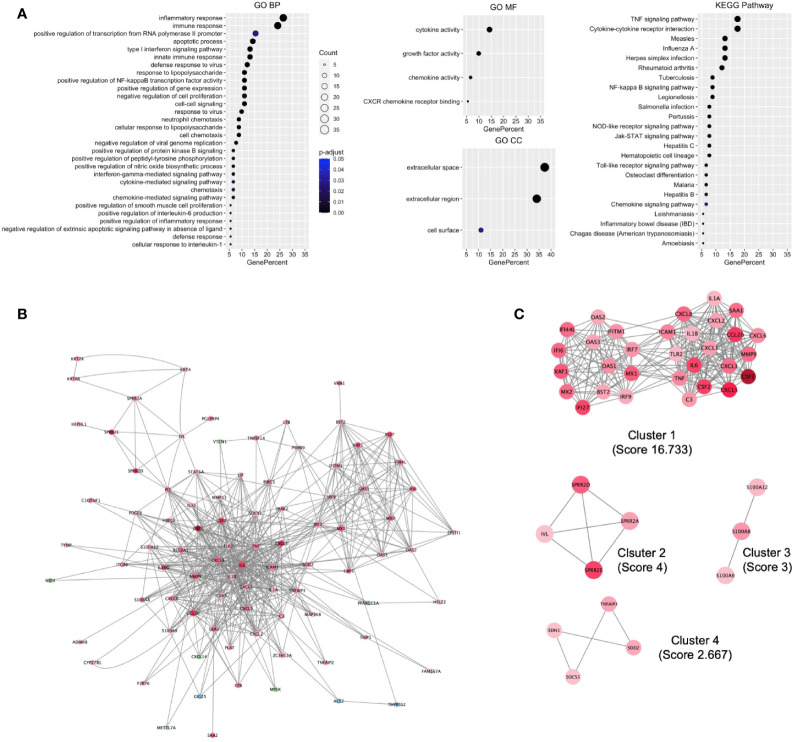
Functional enrichment of host genes in cells infected with SARS-CoV-2 and their biological network. **(A)** GO and KEGG pathways functional enrichment of up-regulated host genes. **(B)** SARS-CoV-2 induced the Host gene regulatory network (*SiHgrn*). **(C)** MCODE clusters extracted from the *SiHgrn* network. MCODE score is given in the bracket. A red node and a green node represent an up-regulated and down-regulated gene, respectively, in DEHGs, while the blue node represents without expression value. GO, Gene Ontology; BP, Biological Processes; MF, Molecular Function; CC, Cell Component; KEGG, Kyoto Encyclopedia of Genes and Genomes.

To understand the biological processes and key pathways altered in SARS-CoV-2–infected cell, the function and pathway enrichment analysis of the DEHGs were performed. The BP of GO analysis revealed that the up-regulated genes are primarily associated with inflammatory response, immune response, apoptotic process, type 1 interferon (IFN) signaling pathway, innate immune response, defense response to viruses, and positive regulation of NFkB transcription factor activity (**Figure 2A**). The MF of GO analysis revealed that the up-regulated genes are primarily associated with cytokine activity, growth factor activity, and chemokine activity (**Figure 2A**); while the CC of GO analysis revealed that the up-regulated genes are significantly related to extracellular space, extracellular region, and cell surface (**Figure 2A**). The KEGG pathway analysis showed the up-regulated genes are significantly enriched in TNF signaling pathway, cytokine-cytokine receptor interaction, measles, influenza A, Herpes simplex infection (**Figure 2A**). The BIOCARTA pathways showed that the up-regulated genes are significantly enriched in signal transduction through IL1R, cytokines and inflammatory response, and cells and molecules involved in the local acute inflammatory response (**Figure S4A**). The DISEASE annotation showed that the up-regulated genes are significantly enriched in type 2 diabetes, ovarian cancer, atherosclerosis, and respiratory syncytial virus bronchiolitis (**Figure S4B**). The BP, MF, and CC of GO, KEGG, BIOCARTA, and DISEASE analysis of down-regulated genes did not show any functional enrichment term. The complete results of GO, KEGG, BIOCARTA, and DISEASE annotation analyses of up-regulated host genes are available in **Table S3**.

### Construction and Analysis of SiHgrn Network

We mapped the 110 genes (108 DEHGs; *ACE2* and *TMPRSS2* of host genes) to the STRING database and retrieved the PPI network having 88 nodes and 563 edges. Then, the PPI network was visualized in Cytoscape software, in which each node represents a protein, while an edge represents an interaction between proteins. Furthermore, the log2FC expression level of DEHGs was integrated into the network, where the red node indicates an up-regulated gene, while the green node indicates a down-regulated gene in SARS-CoV-2–infected cell compared to control and the network is called as *SiHgrn* (**Figure 2B**). In the *SiHgrn network*, 79 nodes are up-regulated and 6 nodes are down-regulated while 3 nodes are not having gene expression level (shown in blue, identified through PPI interaction and not in the list of our DEHGs).

Though *ACE2* and *TMPRSS2* showed no differential expression in the SARS-CoV-2–infected cells compared to control. Interestingly, the *SiHgrn network* revealed that ACE2 interacts with three proteins: IL6 [log2FC=2.92], EDN1 [log2FC=1.06], and TMPRSS2; while TMPRSS2 interacts with two proteins: MX1 [log2FC=2.51] and ACE2. The structural topological of the *SiHgrn network*, including betweenness centrality, closeness centrality, clustering coefficient, and degree, was analyzed with Cytoscape plug-in “NetworkAnalyzer”. The complete result of network topology is given in **Table S4**. The degree of node connectivity is used to determine the node size in the *SiHgrn network*.

### Identification and Analysis of Sub-Network in SiHgrn

Detailed analysis of the *SiHgrn network* with Cytoscape plug-in MCODE identified four local sub-networks (**Figure 2C**; **Table 2**). Among them, only the highest MCODE score, 16.7, *Cluster 1* containing 31 genes, was selected for further study. Highly connected hub genes could influence the topology of a network and biological function. It was found that IL6 and TNF nodes have the highest degree of connectivity [degree=50] in the ***SiHgrn*** *network.* Other top-five hub nodes with degree of connectivity are CXCL8 [44], IL1B [40], MMP9 [35], TLR2 [34], and ICAM1 [32] (**Table S4**). Interestingly, these all top hub genes are highly interconnected, and belong to MCODE *Cluster 1* (**Figure 2C**; **Table 2**).

**Table 2:** List of MCODE clusters and their associated proteins identified from *SiHgrn*.

Cluster	Score (Density*#Nodes)	# Nodes	# Edges	Node IDs
1	16.733	31	251	IL6, CXCL3, CXCL5, IRF9, SAA1, OAS3, CSF2, IFI6, OAS2, CSF3, IRF7, ICAM1, CXCL2, MX1, OAS1, MMP9, IL1A, IL1B, C3, TLR2, IFI27, CXCL6, CXCL1, CCL20, XAF1, TNF, IFI44L, MX2, BST2, IFITM1, CXCL8
2	4	4	6	SPRR2A, IVL, SPRR2D, SPRR2E
3	3	3	3	S100A8, S100A9, S100A12
4	2.667	4	4	SOD2, SOCS3, TNFAIP3, EDN1

**Figure 3:**
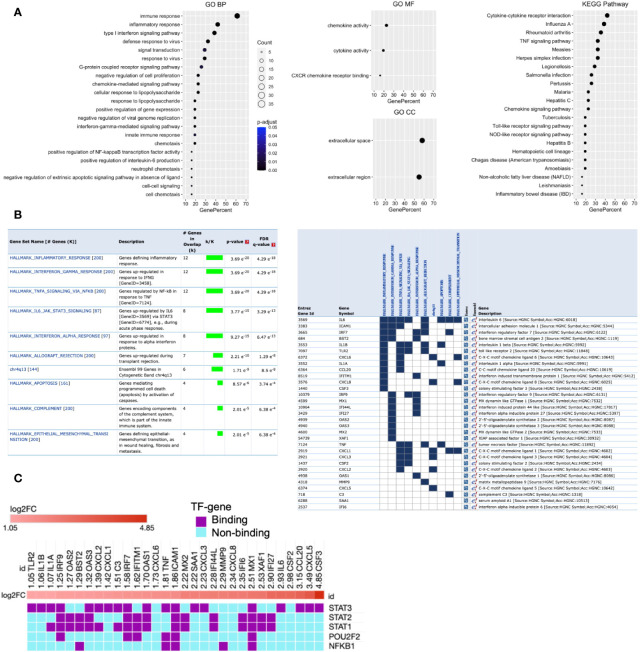
Functional enrichment of *Cluster 1* and its potential upstream regulators. **(A)** GO and KEGG pathways functional enrichment. **(B)** MSigDB Hallmark and positional gene sets enrichment. **(C)** Potential upstream regulators of *Cluster 1* genes. Each column indicates the gene of *Cluster 1*, while each row indicates TF identified by iRegulone. Up-regulated DEHGs in the cluster are red with positive log2FC. TF binding with the mRNA is in purple, while non-binding in cyan.

Function enrichment analysis of *Cluster 1* genes with the BP of GO showed that they are primarily associated with immune response, inflammatory response, type 1 IFN signaling pathway, and defense response to viruses (**Figure 3A**). The MF of GO analysis revealed that *Cluster 1* genes are primarily associated with chemokine activity, cytokine activity, and CXCR chemokine receptor binding (**Figure 3A**). The CC of GO of *Cluster 1* genes are significantly related to extracellular space and extracellular region (**Figure 3A**). The KEGG pathway analysis showed that *Cluster 1* is significantly enriched in cytokine-cytokine receptor interaction, influenza A, rheumatoid arthritis, TNF signaling pathway, measles, Herpes simplex infection (**Figure 3A**). The BIOCARTA pathways showed that the *Cluster 1* genes are significantly enriched in cytokines and inflammatory response, cells and molecules involved in the local acute inflammatory response, and adhesion and diapedesis of granulocytes (**Figure S5A**). The DISEASE annotation showed that the *Cluster 1* genes are significantly enriched in ovarian cancer, respiratory syncytial virus bronchiolitis, asthma, and type 2 diabetes (**Figure S5B**). The complete results of GO, KEGG, BIOCARTA, and DISEASE annotation analyses of *Cluster 1* genes are available in **Table S5**.

Furthermore, enrichment analysis with MSigDB hallmark gene sets showed that *Cluster 1* genes are significantly enriched in several crucial pathways including, inflammatory response, genes up-regulated in response to IFN-γ and IFN-α, genes regulated by NFkB in response to TNF, genes up-regulated by IL6 *via* STAT3, genes up-regulated during transplant rejection and others (**Figure 3B**). While positional gene sets enrichment analysis showed that *Cluster 1* genes are enriched with the location of Cytogenetic Band chr4q13 (Ensembl 99). The detailed information with the statistically significant test associated overlap matrix is presented in the **Table S6**. In addition, the gene set of *Cluster 1* was investigated to identify the gene family according to their protein homology or biochemical activity with MSigDB. Out of 31 genes, 15 genes (*C3, CCL20, CSF2, CSF3, CXCL1, CXCL2, CXCL3, CXCL5, CXCL6, CXCL8, IL1A, IL1B, IL6, SAA1*, and *TNF*) belong to Cytokines and growth factors; 2 genes (*IRF7* and *IRF9*) belong to transcription factors; 4 genes (*BST2, ICAM1, IFITM1, TLR2*) belong to Cell differentiation markers (**Table S7**).

### Analysis for Upstream Regulator of Cluster 1

Transcription factors rapidly modulate gene expression, especially in activating the host immune response against viral infection. Their identification will improve the understanding of the immunoregulatory mechanism, and a better approach to control the cytokine storm and viral defense in severe COVID-19. Therefore, we analyzed the *Cluster 1* to determine its potential upstream TFs regulators using the iRegulone tool. The output result showed that most of the *Cluster 1* genes, up-regulated (log2FC >1) in SARS-CoV-2 response, are under the control of TFs STAT1, STAT2, STAT3, POU2F2, and NFkB1 (**Figure 3C**).

### Biological Pathways Enrichment of “*Host Response Signature Network*”

**Figure 4:**
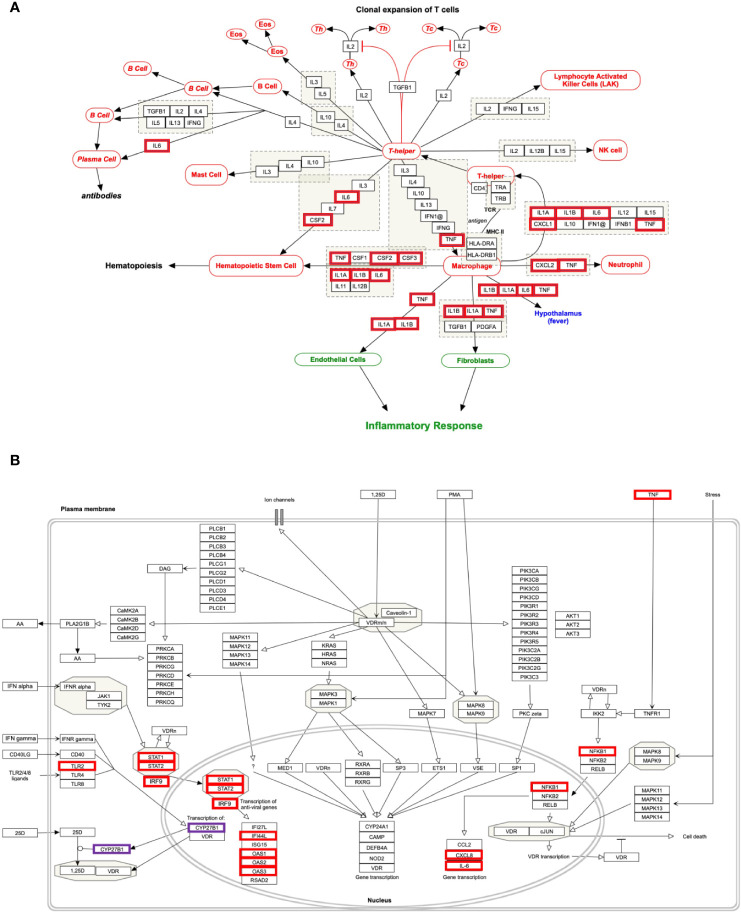
Biological pathways enrichment of “*host response signature network*”: **(A)** “Cytokines and Inflammatory Response” and **(B)** “Non-genomic actions of 1,25 dihydroxyvitamin D3”. Gene from the “*host response signature network*” of the SARS-CoV-2 infected cell is in the red box. The IRF9 and CYP27B1 genes are up-regulated in DEHGs. CYP27B1 in the purple box is not associated with the “*host response signature network”*.

**Figure 5:**
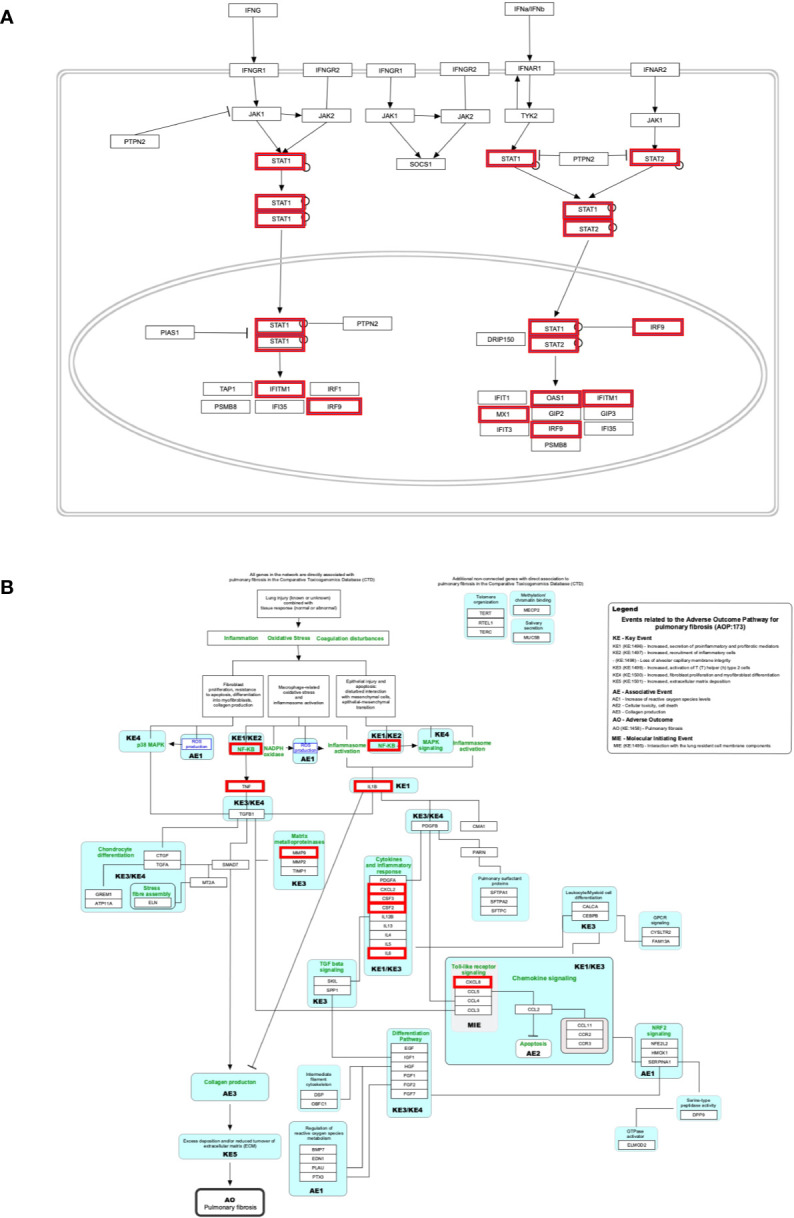
Biological pathways enrichment of “host signature network”: **(A)** “The human immune response to tuberculosis” and **(B)** “Lung fibrosis.” Gene from the “*host response signature network*” of the SARS-CoV-2 infected cell is in the red box.

The genes of the “*host response signature network”* (consist of *Cluster 1* and its upstream regulators =36 query genes) were analyzed with NDEx, which returns a list of relevant pathways according to the highest similarity score. Among them, a list of top nine pathways with more than 5 overlapping genes with our query genes was selected (**Table 3**). It was observed that the “Cytokines and Inflammatory Response” pathway significantly matched with the “*host response signature network*” with the highest similarity score [similarity score=0.21; the number of overlap gene=8; p-value=2.70e-10] (**Table 3** and **Figure 4A**). On the other hand, “Non-genomic actions of 1,25 dihydroxyvitamin D3” pathway significantly matched with the “*host response signature network*” with the highest number of genes [similarity score=0.19; the number of overlap gene=11; p-value=1e-12] (**Table 3** and **Figure 4B**). **Figure 4B** showed that TNF induced NFkB1 signaling pathway is enriched with four proteins TNF, NFkB1, CXCL8, and IL6. While, IFN-α induced Jak-STAT signaling pathway is enriched with proteins STAT1, STAT2, ILI44L, OAS1, OAS2, and OAS3. It was also observed that the gene of TLR2 is over-expressed in our study, which involves inducing the expression of *CYP27B1* and *VDR* genes. In our study, *VDR* is not differentially expressed; but, *CYP27B1* is up-regulated [log2FC=1.21] in the SARS-CoV-2–induced cells (**Table S1**). Interestingly, the *SiHgrn network* shows interactions of CYP27B1 with IL6 and TLR2 (**Figure 2B**). Despite this, CYP27B1 is not associated with any of the MCODE clusters (**Figure 2C**)*. *The genes of “*host response signature network*” are also showing enrichment with other pathways involve in immune response including “the human immune response to tuberculosis” [similarity score=0.20; the number of overlap gene=6; p-value=1.57e-8] (**Table 3** and **Figure 5A**); “Photodynamic therapy-induced NF-kB survival signaling” [similarity score=0.17; the number of overlap gene=9; p-value=1e-12] **(** **Table 3** and **Figure S6A**); “IL-10 Anti-inflammatory Signaling Pathway” [similarity score=0.16; number of overlap gene=6; p-value=4.02e-12] (**Table 3** and **Figure S6B**); “Type II interferon signaling (IFN-γ)” [similarity score=0.13; the number of overlap gene=6; p-value=3.09e-7] (**Table 3** and **Figure S6C**); “IL27-mediated signaling events” [similarity score=0.11; the number of overlap gene=6; p-value=3.72e-10] (**Table 3** and **Figure S7A**); and “IL23-mediated signaling events” [similarity score=0.11; the number of overlap gene=7; p-value=8.21e-11] (**Table 3** and **Figure S7B**). Interestingly, genes of the “*host response signature network*” are also showing enrichment with the “lung fibrosis” pathway [similarity score=0.12; the number of overlap gene=8; p-value=9.35e-11], which could indicate the etiology of lung damage in COVID-19 patients (**Table 3** and **Figure 5B**).

**Table 3:** Biological pathways enrichment of “*host response signature network*” using NDEx v2.4.5.

Pathway Name	Pathway Properties	Number of overlap gene	p-value <	Similarity score
WP530—Cytokines and Inflammatory Response - Homo sapiens http://identifiers.org/wikipathways/WP530_r96982 Source: wikipathways	Nodes:124; Egde:37	8 genes	2.70e-10	0.21
WP4341—Non-genomic actions of 1,25 dihydroxyvitamin D3 - Homo sapiens http://identifiers.org/wikipathways/WP4341_r107169 Source: WikiPathways	Nodes:166; Egde:68	11 genes	1e-12	0.19
WP4197—The human immune response to tuberculosis - Homo sapiens http://identifiers.org/wikipathways/WP4197_r105840 Source: wikipathways	Nodes:68; Egde:30	6 genes	1.57e-8	0.20
WP3617—Photodynamic therapy-induced NF-kB survival signaling - Homo sapiens http://identifiers.org/wikipathways/WP3617_r106541 Source WikiPathways	Nodes: 59; Egde:12	9 genes	1e-12	0.17
WP4495—IL-10 Anti-inflammatory Signaling Pathway - Homo sapiens http://identifiers.org/wikipathways/WP4495_r102692 Source WikiPathways	Nodes: 40; Egde:17	6 genes	4.02e-12	0.16
WP619—Type II interferon signaling (IFN*-*γ) - Homo sapiens http://identifiers.org/wikipathways/WP619_r106442 Source WikiPathways	Nodes: 127; Egde:83	6 genes	3.09e-7	0.13
WP3624—Lung fibrosis - Homo sapiens http://identifiers.org/wikipathways/WP3624_r106633 Source: WikiPathways	Nodes: 186; Egde:55	8 genes	9.35e-11	0.12
IL27-mediated signaling events Source: Pathway Interaction Database (PID) curated by NCI/Nature.	Node: 26; Edge: 101	6 genes	3.72e-10	0.11
IL23-mediated signaling events Source: Pathway Interaction Database (PID) curated by NCI/Nature.	Node: 37; Edge: 201	7 genes	8.21e-11	0.11

### Validation of Host Response Pathways Using Gene Expression Data

To validate the identified host response pathways, a list of differentially expressed host genes was taken from a recent study conducted in 24 patients who died from COVID-19 (37). The study used the lung autopsy specimens from the 25 samples from the high viral load and 21 samples from the low viral load patients. The study identified 338 up-regulated and 5,710 down-regulated host genes in the high viral load samples compared to low viral load samples (|log2FC| >0.3 and adjusted p-value <0.01), and we referred it to as DEHGs^COVID-19^ (37). Different analytical techniques, including viral gene expression, were considered for the classification of COVID-19 patients into the high and low viral load. The SARS-CoV-2 gene *ORF1a* is over-expressed (log2FC=9.27, adjusted p-value= 2.68e-25) in the high viral load samples compared to the low viral load samples. With the NDEx tool (28 September 2020), the up-regulated genes of DEHGs^COVID-19^ identifies the “response to interferon-alpha (GO:0035455)” as a top scores relevant pathway with the similarity score=0.12; the number of overlap gene=8 (*ADAR*, *BST2*, *EIF2AK2*, *IFIT2*, *IFIT3*, *IFITM1*, *IFITM3*, *TPR*); and p-value=2.27e-6. After that, the DEHGs^COVID-19^ was mapped to previously identified host response pathways with NDEx. The up-regulated and down-regulated genes with their associated pathways are given in **Table 4**, and the gene expression level is provided in **Table S8**.

**Table 4:** The differentially expressed host genes (DEHGs^COVID-19^) shows the overlapping with host response pathways using NDEx v2.4.5.

Pathway Name	Up-regulated gene	Down-regulated gene
WP530—Cytokines and inflammatory response	No gene	*IL1B*, *IL12B*, *TNF*, *IL6*
WP4341—Non-genomic actions of 1,25 dihydroxyvitamin D3	*STAT1*, *STAT2*, *IFI44L*, *ISG15*, *OAS1*, *OAS2*, *OAS3*, *RSAD2*, *PIK3R1*, *PIK3C2A*	*CAMK2B*, *VDR*, *TNF*, *CYP27B1*, *CYP24A1*, *CXCL8*, *IL6*, *CAMK2A*, *PRKCB*, *RXRA*, *PRKCA*, *PIK3C2B*, *MAPK13*
WP4197—The human immune response to tuberculosis	*STAT1*, *STAT2, JAK2, PSMB8, TAP1, IFI35, IFIT1, IFITM1, IFIT3, OAS1, MX1*	No gene
WP3617—Photodynamic therapy-induced NF-kB survival signaling	No gene	*IL1B*, *TNF*, *MMP9*, *IL6*, *PTGS2*
WP4495—IL-10 anti-inflammatory signaling pathway	*STAT1, STAT2*	*TNF*, *IL6*, *HMOX1*
WP619—Type II interferon signaling (IFN*-*γ)	*IFIT2*, *STAT1*, *STAT2*, *JAK2*, *EIF2AK2*, *OAS1*, *HLA-B*, *GBP1*, *TAP1*	*IL1B*
WP3624—Lung fibrosis	*MT2A* and *EDN1*	*IL1B*, *CCR3*, *CXCL8*, *IL12B*, *TNF*, *CYSLTR2*, *FGF1*, *IL6*, *BMP7*, *MMP9*, *MUC5B*, *SFTPC*, *MECP2*, *TGFA*, *HMOX1*
IL27-mediated signaling events	*JAK2, STAT1, STAT2*	*IL1B*, *IL12B*, *TNF*, *IL6*
IL23-mediated signaling events	*JAK2, PIK3R1, STAT1*	*IL1B*, *IL12B*, *TNF*, *IL6*

**Figure 6:**
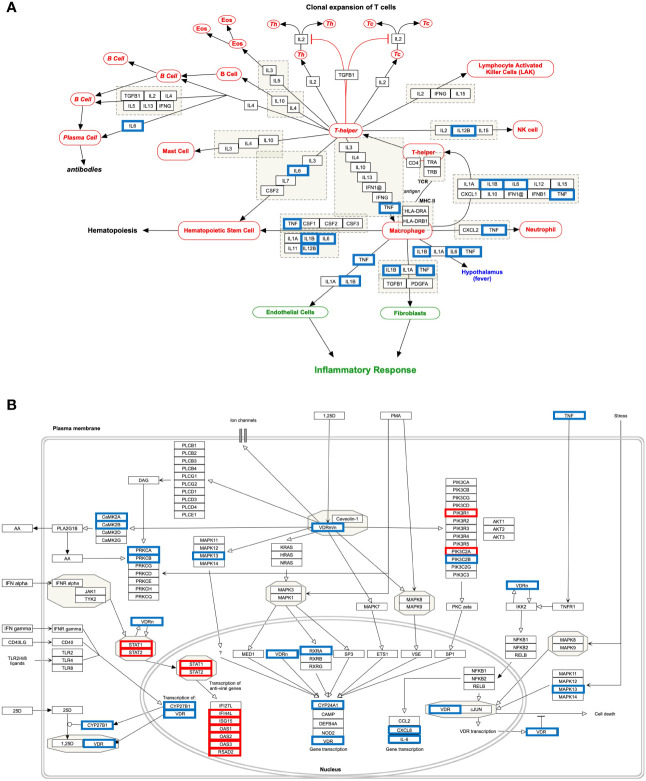
Host response pathways using differentially expressed host genes (DEHGs^COVID-19^) in the high viral load compared to the low viral load of lungs autopsy of COVID-19 patients. **(A)** “Cytokines and Inflammatory Response” and **(B)** “Non-genomic actions of 1,25 dihydroxyvitamin D3.” A red box indicates an up-regulated gene, while a blue box represents a down-regulated gene in the high viral load compared to the low viral load of COVID-19 lung samples, respectively.

Our analysis observed that only 4 down-regulated genes (*IL1B*, *IL12B*, *TNF*, and *IL6*) were associated with “Cytokines and Inflammatory Response” (**Table 4** and **Figure 6A**). However, 10 up-regulated genes (*STAT1*, *STAT2*, *IFI44L*, *ISG15*, *OAS1*, *OAS2*, *OAS3*, *RSAD2*, *PIK3R1*, *PIK3C2A*) and 12 down-regulated genes (*CAMK2B*, *VDR*, *TNF*, *CYP27B1*, *CYP24A1*, *CXCL8*, *IL6*, *CAMK2A*, *PRKCB*, *RXRA*, *PRKCA*, *PIK3C2B*, *MAPK13*) were associated with the “Non-genomic actions of 1,25 dihydroxyvitamin D3” pathway (**Table 4** and **Figure 6B**). The 11 up-regulated genes (*STAT1*, *STAT2, JAK2, PSMB8, TAP1, IFI35, IFIT1, IFITM1, IFIT3, OAS1, MX1*) were mapped to “the human immune response to tuberculosis” pathway (**Table 4** and **Figure S8A**). Furthermore, two up-regulated genes (*MT2A* and *EDN1*) and 15 down-regulated genes (*IL1B*, *CCR3*, *CXCL8*, *IL12B*, *TNF*, *CYSLTR2*, *FGF1*, *IL6*, *BMP7*, *MMP9*, *MUC5B*, *SFTPC*, *MECP2*, *TGFA*, *HMOX1*) were associated with “lung fibrosis” pathway (**Table 4** and **Figure S8B**). The DEHGs^COVID-19^ associated with others pathways are also provided in the supplementary figures, including “Photodynamic therapy-induced NF-kB survival signaling” (**Table 4** and **Figure S9A**); “IL-10 Anti-inflammatory Signaling Pathway” pathway (**Table 4** and **Figure S9B**); “Type II interferon signaling (IFN-γ)” pathway (**Table 4** and **Figure S9C**); “IL27-mediated signaling events” (**Table 4** and **Figure S10A**); and “IL23-mediated signaling events” (**Table 4** and **Figure S10B**).

## Discussion

In the present work, *omics* data and computational methods were used to understand the underlying mechanisms of SARS-CoV-2 induced altered host gene regulatory networks, and the potential regulatory mechanism of Vitamin D in suppressing cytokine storm and reducing viral load. Using the gene expression data, this study identified 108 DEHGs, including 93 up-regulated and 15 down-regulated genes in SARS-CoV-2 infected NHBE cell compared to control. The up-regulated genes are significantly enriched in the inflammatory response, immune response that induces through TNF signaling pathways, and cytokine-cytokine receptor interactions (**Figure 2A**). In order to understand the regulatory network, the DEHGs were integrated with the human PPI to generate a *SiHgrn network* (**Figure 2B**), which contains a highly connected sub-network of *Cluster 1* (**Figure 2C**). The functional enrichment analysis of *Cluster 1* showed that its primary role in immune response and inflammatory response, which induces through the cytokine-cytokine receptor interactions pathway (**Figures 3A, B** **)**. Besides, enriched gene percentages in *Cluster 1* was increased compared to that of the up-regulated genes of DEHGs, which indicates that *Cluster 1* plays a significant role in inducing an inflammatory response in COVID-19 (**Figures 2A**, **3A**; **Figures S4**, **S5**). Furthermore, about half of *Cluster 1* genes belong to the family of cytokines and growth factors, which trigger a severe inflammatory response (**Table S7**).

Additional analysis of *Cluster 1* with the iRegulone tool identified five potential upstream regulators: STAT1, STAT2, STAT3, POU2F2, and NFkB1 (**Figure 3C**). Furthermore, the up-regulation of *STAT1* and *STAT2* in the high viral load lung samples compared to the low viral load lung samples of DEHGs^COVID-19^ supports our finding of *Cluster 1* upstream regulators (**Figures 3C**, **6B**).The *Cluster 1* and these five upstream regulators are collectively referred to as “*host response signature network*”, which was used for the detailed understanding of the regulatory mechanism of cytokine storm, host immune response, and clinical manifestation in COVID-19. Functional enrichment analysis of the “*host response signature network*” with the NDEx tool revealed several alterations in crucial pathways associated with SARS-CoV-2 infection and pathogenesis (**Table 3**).

### The “Cytokines and Inflammatory Response” Pathway

Our analysis showed that the “*host response signature network*” is significantly associated with “cytokines and inflammatory response” pathway (**Table 3** and **Figure 4A**). The gene of cytokine IL6, TNF, IL1A, IL1B, CSF2, and CSF3 were over-expressed in SARS-CoV-2 infected cells and could be responsible for proliferating hematopoietic stem cells to fight against the virus (**Figure 4A**). Other cytokine genes were also over-expressed in SARS-CoV-2 infected cells, including TNF, IL1A, and IL1B, which activate the fibroblasts and endothelial cells for inflammatory response. **Figure 4A** showed that CXCL2 and TNF induce neutrophil, while IL1A, IL1B, IL6, CXCL1, and TNF induce the T-helper cell. Enhance concentration of IL1A, IL1B, IL6, and TNF cause increase body temperature *via* the hypothalamus in COVID-19 (**Figure 4A**).

The binding of CSF2 to its receptor on the myeloid cell induces its differentiation and proliferation into monocytes and macrophages (38). However, another study showed that CSF2 required for normal pulmonary physiology, including surfactant homeostasis in the mice (39, 40). Disruption of CSF2 resulted in pulmonary alveolar proteinosis due to compromise in the development of alveolar macrophage, making lungs susceptible to infection (39, 40). During infection, CSF3 works along with IL3, IL6, and CSF2 to stimulate neutrophil granulopoiesis in the bone marrow to restore neutrophil homeostasis. The expression of CSF3 is higher in poorly controlled asthma, and inhibiting the signaling by neutralizing its receptor, CSF3R, decrease the production of mucus and hyperreactivity in the airway (41).

An effective immunological response against SARS-CoV-2 infection requires an appropriate cytokine level and an adequate number of activated T cells for reducing the viral load. A prior study found that the number of T cells was drastically decreased, while the level of TNF, IL6, and IL10 were significantly increased in severe COVID-19 patients compared to healthy control (42). Besides, the authors observed that the concentration of TNF, IL6, and IL10 was negatively correlated with the number of T cells, including total T cell, CD4+ cell, and CD8+ cells. Hence, they suggested that these cytokines play a critical role in the acute inflammatory response and lower the survival of T cells. Furthermore, the survived T cells became functionally exhausted in COVID-19, as indicated by the expression of PD-1 and Tim-3 on the T cell surface (42). The cells of healthy mice, including epithelium, endothelium, and fibroblasts, release different cytokines and involve in the immune response against pathogens (43). Our study found that several cytokine genes are over-expressed in NHBE cells infected with SARS-CoV-2. Thus, suggesting that this NHBE cell is also involved in the immunoregulatory role and is strongly implicated in the immune response against SARS-CoV-2 infection (**Figure 2A** and **Table S3**), and therefore support previous findings (42, 43).

### The “Non-genomic Actions of 1,25 Dihydroxyvitamin D3 (1,25 D)” Pathway

The 1,25 D is a biologically active form of vitamin D. Interestingly; prior studies observed that lower Vitamin D is associated with increased risk of autoimmune disease, inflammation, bacterial and viral infection including SARS-CoV-2 (12–15). Furthermore, an adequate amount of vitamin D could substantially reduce the risk of cytokine storm, other complications, and death in COVID-19 patients; however, its underlying mechanism is not clearly known (14, 17, 44).

Our analysis showed that the “*host response signature network*” is significantly associated with non-genomic actions of 1,25 D pathway with p-value 1e-12 (**Table 3** and **Figure 4B**). Viral infection recognized by innate immune systems triggers two signaling cascades: (a) stimulating the production of pro-inflammatory cytokine (e.g., IL1, IL6, TNF) through NFkB1-mediated pathway; (b) stimulating the production of type I and type III IFNs through interferon regulatory factor (IRF3 and IRF7) mediated pathways (45). Based upon data analysis and literature mining, our study proposed the following two pathways through which Vitamin D could reduce the cytokine storm and enhance the antiviral response.

#### TNF-Induced NFkB1 Signaling Pathway

NFkB is a family of transcription factors composed of five proteins: (i) NFkB1 (p50 and its precursor p105), (ii) NFkB2 (p52 and its precursor p100), (iii) RelA (p65), (iv) RelB, and (v) c-Rel. These proteins interact with each other to form different types of homo- and hetero-dimers and regulate important biological processes, including inflammation, immunity, and apoptosis. In an unstimulated cell, NFkB1 dimer is sequestered in the cytoplasm through physical association with NFkB1 inhibitory protein (IkB). The TNF, IL1, LPS, or various other external stimuli induce the canonical signal transduction pathway of NFkB1 (46). Activation of this pathway involves site-specific phosphorylation of IKKβ and formation of an active kinase complex IKK, which subsequently phosphorylates and degrades the downstream target IkB. This leads to the release and translocation of NFkB1 to the nucleus and activates the target gene transcripts involved in immune response, including *IL6* and *CXCL8* (**Figure 4B**). The VDR binds to IKKβ and prevents its phosphorylation and formation of IKK, and thus inhibit the NFkB1 activation (47). The 1,25 D binds with VDR and enhances the interaction between VDR-IKKβ; thus, a study concluded that 1,25 D blocks the TNF induced NFkB1 activation (47). Our analysis found that NFkB1 regulates the expression of *IL6* and *CXCL8* (*IL8*, with the lowest adjusted p-value), which are over-expressed in SARS-CoV-2 infected cells (**Figure 4B**). High levels of IL6 and CXCL8 are associated with severe cases of COVID-19 pathogenesis. Collectively, the data suggest that the active form of Vitamin D could prevent the translocation of NFkB1 to the nucleus and, consequently, inhibit the cytokine storm in COVID-19.

#### IFN-α–Induced Jak-STAT Signaling Pathway

The IFN has an essential role in the innate immune response against viruses. Viral infection in human activates type 1 IFNs (IFN-α, IFN-β, IFN-ϵ, IFN-κ, IFN-ω), type II IFN (IFN-γ), and type III IFN (IFN-λ) (45). IFN-α binds to the cell receptor and activates the JAK1 and Tyk2, which subsequently phosphorylate the downstream targets STAT1 and STAT2 (48). The phosphorylation causes the dimerization of STAT1 with STAT2, which associates with IRF9 to forms a major transcription factor ISGF3 complex. The activation and translocation of ISGF3 from the cytoplasm to the nucleus induces interferon-stimulated genes (ISGs) that provide the antiviral activity of a cell (49).

Prior studies showed that Vitamin D deficiency had a poor response of IFN-α based therapy on chronic hepatitis C virus (HCV) (50, 51). In Huh-7.5 cells, vitamin D inhibited the production of infectious HCV in a dose-dependent manner (50). A subsequent study revealed that a combination of 1,25 D with IFN-α increases the inhibitory effect on HCV replication (52). Furthermore, the study observed a constitutive inhibitory interaction between VDR with STAT1 in Huh-7.5 cells infected with HCV; however, this interaction decreased after cells were treated with IFN-α alone and completed abolished when cells were treated with both 1,25 D and IFN-α. Consequently, STAT1 dissociated from VDR and formed the ISGF3, which moved to the nucleus for inducing its target genes (52). Furthermore, the study demonstrated that the silencing of VDR expression in cells and then treated with IFN-α resulted in a significantly more potent induction of mRNA expression of ISGs (*IFI27L* and *IFI44L*) than the control cells. Thus the study concluded that VDR is an inhibitor of IFN-α induced signaling through the Jak–STAT pathway (52).

Our study indicates that the high expression of *IRF9* in the SARS-CoV-2 infected cells could be involved in the formation of transcription factor ISGF3, which subsequently increases the expression of ISGs. The genes *ILI44L*, *OAS1*, *OAS2*, and *OAS3*, whose expression are under the control of ISGF3, were over-expressed in SARS-CoV-2 infected cells compared to control (**Figure 4B**). Furthermore, this study showed over-expression of *TLR2* and *CYP27B1* in SARS-CoV-2 infected cells compared to control. A prior study demonstrated that 1,25 D supplement enhances the TLR-mediated macrophage ability to fight against Mycobacterium tuberculosis (53). The *CYP27B1* gene encodes an enzyme 1α-hydroxylase that converts vitamin D to its active form, 1,25 D. A previous study showed that insufficient Vitamin D and polymorphism in promotor of *CYP27B1*-1260 have been associated with chronic HCV infection as well as a poor response to IFN-α based therapy (50, 51, 54). Furthermore, *IRF7* is up-regulated in both DEHGs and DEHGs^COVID-19^ SARS-CoV-2 infected samples. A recent study identified the loss-of-function mutation at the loci of TLR3 and IRF7 in severe patients with influenza and COVID-19, resulting in preventing type 1 IFN production, thus emphasizing the significance of type 1 IFN in controlling virus production (55).

A study used transcription and serum profiling of COVID-19 patients and revealed that SARS-CoV-2 infection induces a high level of chemokines and pro-inflammatory cytokines such as IL6, while very low level of IFN-I or IFN-III resulting limited antiviral ISGs response (27). Accumulating finds suggested that an imbalance between a high level of pro-inflammatory cytokines production and a low IFN response could cause severe COVID-19 pathogenesis (27, 45). Based on our findings, we expect that a high level of vitamin D could have two consequences: (i) down-regulation of cytokine storm, and (ii) up-regulation of ISGs for a robust antiviral response. Analysis of differentially expressed host genes from high viral load compared to low virus load, DEHGs^COVID-19^, demonstrated the crucial role of “Cytokines and Inflammatory Response” and “Non-genomic actions of 1,25 dihydroxyvitamin D3” pathways in COVID-19. It also revealed that *VDR* and pro-inflammatory genes are down-regulated, while *STAT1*, *STAT2*, and interferon response genes are up-regulated in the high viral load lung samples compared to the low viral lung samples of COVID-19 (**Table 4** and **Figures 6A, B** **)**. Desai *et al*. reported that the COVID-19 patients classified as high viral load induced more interferon response pathway genes for antiviral defense programs, resulting in a significantly short duration of illness than patients with low viral load (37). A recent study showed that human epithelial cells infected with SARS-CoV-2 induces a high interferon response *via* Jak-STAT signaling pathway, which controls the viral replications and *de novo* virus production (56) Another study observed that type I IFNs suppress the SARS-CoV-2 activities in cultured cells, showing the potency of type I IFNs to treat COVID-19 (57).

Therefore, based upon accumulating evidence and gene expression data of SARS-CoV-2 infected samples, our finding revealed that an adequate level of Vitamin D binds with VDR that could results. (i) minimizes the expression of pro-inflammatory cytokines by blocking the TNF induced NFkB1 signaling pathway, and (ii) induces the expression of ISGs for antiviral defense through activating the IFN-α induced Jak-STAT signaling pathway for reducing the virus load.

### The “Human Immune Response to Tuberculosis” Pathway

Only 10-20% of people infected with *Mycobacterium tuberculosis* have a lifetime risk of showing signs called “active” tuberculosis (TB) (58, 59). Pathogens responsible for both COVID-19 and TB show high similarity regarding its transmission mode and symptoms, including infecting the lungs, having fever, cough, and shortness of breath. A study observed that countries implemented mass vaccination programs for the Bacille Calmette-Guérin vaccine (BCG) against TB showed a significant reduction in COVID-19 mortality than those who never applied it (60). Thus, the authors suggested that BCG vaccination could protect people from COVID-19; however, the experimental evidence and underlying molecular mechanism are still lacking (60). IFN-γ is a crucial cytokine produced by CD4^+^ T cells and activates macrophage to providing resistance to TB infection (61). Low plasma concentration of IFN-γ was associated with active TB infection (62). Moreover, studies identified that a single nucleotide polymorphism (+874T/A) at the first intron of *IFN-γ* increases the chance to develop active TB (62, 63). A study analyzed the blood transcriptome data and identified over-expression of IFN I and IFN II inducible genes, including *IFITM1*, *IRF1*, *IRF9*, *OAS1*, *MX1*, *STAT1*, and *STAT2* **(** **Figure 5B**), also identified up-regulated inflammatory genes in the neutrophils of active TB compared to healthy control (26). In contrast, the expression of T and B-cell specific genes were down-regulated in active tuberculosis (26, 58). In the current study, six genes (*IFITM1*, *IRF9*, *MX1*, *OAS1*, *STAT1*, and *STAT2*) of the “*host response signature network*” are significantly overlapping with the “human immune response to tuberculosis” pathway with p-value 1.57e-8 (**Table 3** and **Figure 5B**). Furthermore, 11 genes (*STAT1*, *STAT2, JAK2, PSMB8, TAP1, IFI35, IFIT1, IFITM1, IFIT3, OAS1*, and *MX1*) were up-regulated in the “human immune response to tuberculosis” pathways in the high viral load compared to the low viral load COVID-19 samples (**Table 4** and **Figure S8A**). Overlapping of activated genes and pathways between COVID-19 and active TB indicates both diseases are mechanistically related, which might be the reason that BCG vaccination could protect people from severe COVID-19 (60).

### The “Lung Fibrosis” Pathway

The complication of COVID-19 includes the development of lung fibrosis, excessive deposition of collagen and extracellular matrix (EM), destroying normal lung architecture, resulting in difficulty breathing and lung failure (64). The ARDS is considered as one of the significant factors for the lung fibrosis (64). A prior study demonstrated that SARS-CoV-1 infection promotes lung fibrosis by enhancing the TGF-β signaling and reducing the ACE2 expression (65, 66). ACE2 receptor, responsible for SARS-CoV-1 and SARS-CoV-2 infection, is primarily involved in the degradation and clearance of ANG-II. Reducing the clearance of ANG-II induces extracellular matrix deposition and lung fibrosis (66).

Our analysis showed that eight genes (*CSF2*, *CSF3*, *CXCL2*, *CXCL8*, *IL1B*, *IL6*, *MMP9*, and *TNF*) are significantly overlapping with the lung fibrosis pathway with p-value 9.35e-11 (**Table 3** and **Figure 5B**). Furthermore, 15 genes (*IL1B*, *CCR3*, *CXCL8*, *IL12B*, *TNF*, *CYSLTR2*, *FGF1*, *IL6*, *BMP7*, *MMP9*, *MUC5B*, *SFTPC*, *MECP2*, *TGFA*, *HMOX1*) were up-regulated in the “lung fibrosis” pathways in the low viral load compared to the high viral load COVID-19 samples (**Table 4** and **Figure S8A**). Thus, our study indicates the relationship between SAR-CoV-2 infection and lung fibrosis. This study also supports the previous finding that high levels of NFkB, TNF, SFTPC, MUC5B, and other proteins could promote lung fibrosis (67). Diffuse alveolar damage and lung fibrosis are frequently observed in SARS-CoV-2 patients; however, the underlying mechanism is not clearly understood (37).

## Conclusion

Our study uses bioinformatics and systems biology approaches and identified the SARS-CoV-2 induced altered host gene regulatory sub-network, *Cluster1*, responsible for cytokine storm.

*Cluster 1* contains highly interconnected 31 genes under the regulation of STAT1, STAT2, STAT3, POU2F2, and NFkB1, making a “*host response signature network*”. The association of “*host response signature network*” with “cytokines and inflammatory response”, “non-genomic action of vitamin D”, “the human immune response to tuberculosis”, and “lung fibrosis” indicates that it plays an essential role in COVID-19 pathogenesis.

**Figure 7:**
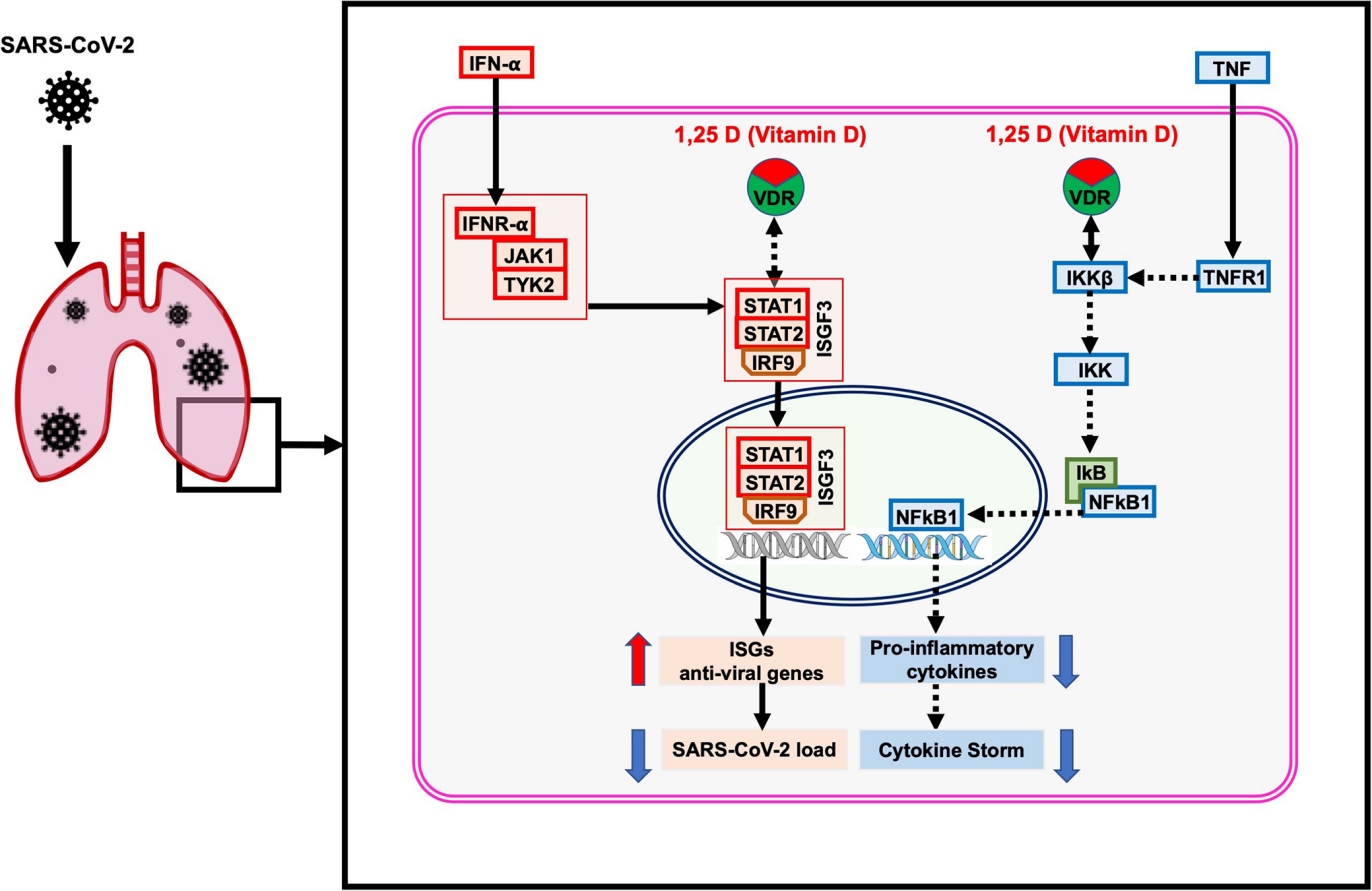
The proposed model of the non-genomic actions of 1,25 dihydroxyvitamin D3 (1,25 D) in the lungs infected with SARS-CoV-2. The 1,25 D is a biologically active form of vitamin D that blocks the TNF induced NFkB1 activation. The 1,25 D binds with VDR and enhances the interaction between VDR-IKKβ, which prevents phosphorylation of IKKβ and formation of active IKK. Therefore, the degradation of IkB is blocked, resulting in preventing the translocation of NFkB1 to the nucleus. Consequently, the transcription and expression of NFkB1 target genes responsible for the cytokine storm are suppressed. In addition, 1,25 D enhances the IFN-α induced Jak-STAT signaling pathway. IFN-α activates the JAK1 and TYK2 signaling, which subsequently phosphorylate and activate the downstream targets STAT1 and STAT2. The 1,25 D binds with VDR and induces the dissociation between VDR-STAT1; thus, STAT1 is available for phosphorylation and formation of active TF complex ISGF3. The translocation of ISGF3 to the nucleus activates the transcription of interferon-stimulated genes (ISGs), which provide antiviral activity and reduce the SARS-CoV-2 load in cells. The figure was adapted from the WikiPathways (WP4341) www.wikipathways.org/instance/WP4341.

Our study revealed that Vitamin D could bind with its receptor and work through two pathways to suppress cytokine storm and reduce viral load (**Figure 7**). Our study has few limitations, including a small sample size of SARS-CoV-2 infected host cells and a lack of experimental validation supporting the identified mechanisms. Furthermore, this study did not include the influence of genetic polymorphism relevant to identified pathways, especially pro-inflammatory and type 1 IFN related signaling pathways on the severity of COVID-19. Therefore, this study proposed an urgent need to check the suitability of Vitamin D in combination with IFN-α to suppress the cytokine storm and reduce viral load in the SARS-CoV-2 infected experimental model. Our current study provides in-depth insight into a better understanding of the regulatory mechanism of cytokine storm and vitamin D; it might be helpful to develop a better approach for therapeutic intervention using vitamin D for severe COVID-19 patients.

## Data Availability Statement

Publicly available datasets were analyzed in this study. This data can be found here: https://www.ncbi.nlm.nih.gov/geo/query/acc.cgi?acc=GSE147507, https://www.ncbi.nlm.nih.gov/geo/query/acc.cgi?acc=GSE150316.

## Author Contributions

FA conceptualized and designed the whole study, performed all the analyses, interpreted the results, wrote, and revised the manuscript.

## Conflict of Interest

The author declares that the research was conducted in the absence of any commercial or financial relationships that could be construed as a potential conflict of interest.
