# Supplementary material for: A Network-Based Analysis Reveals the Mechanism Underlying Vitamin D in Suppressing Cytokine Storm and Virus in SARS-CoV-2 Infection
Source: Front Immunol. 2020 Dec 9;11:590459. doi: 10.3389/fimmu.2020.590459 (PMC7756074; doi:10.3389/fimmu.2020.590459)
Supplement: Supplementary file 7 [file Table_7.docx]

| **Gene families** | **Cytokines and growth factors** | **Transcription factors** | **Homeodomain proteins** | **Cell differentiation markers** | **Protein kinases** | **Translocated cancer genes** | **Oncogenes** | **Tumor suppressors** |
| --- | --- | --- | --- | --- | --- | --- | --- | --- |
| **Tumor suppressors** | 0 | 0 | 0 | 0 | 0 | 0 | 0 | 0 |
| **Oncogenes** | 0 | 0 | 0 | 0 | 0 | 0 | 0 |  |
| **Translocated cancer genes** | 0 | 0 | 0 | 0 | 0 | 0 |  |  |
| **Protein kinases** | 0 | 0 | 0 | 0 | 0 |  |  |  |
| **Cell differentiation markers** | 0 | 0 | 0 | 4 |  |  |  |  |
| **Homeodomain proteins** | 0 | 0 | 0 |  |  |  |  |  |
| **Transcription factors** | 0 | 2 |  |  |  |  |  |  |
| **Cytokines and growth factors** | 15 |  |  |  |  |  |  |  |

**Table S7:** *Cluster 1* genes are categorized into different gene families according to Molecular Signature Database (MSigDB). Value in the box indicates the number of gene associated with family.
