# Supplementary material for: A Network-Based Analysis Reveals the Mechanism Underlying Vitamin D in Suppressing Cytokine Storm and Virus in SARS-CoV-2 Infection
Source: Front Immunol. 2020 Dec 9;11:590459. doi: 10.3389/fimmu.2020.590459 (PMC7756074; doi:10.3389/fimmu.2020.590459)
Supplement: Supplementary file 9 [file Presentation_1.pptx]

## Slide 1
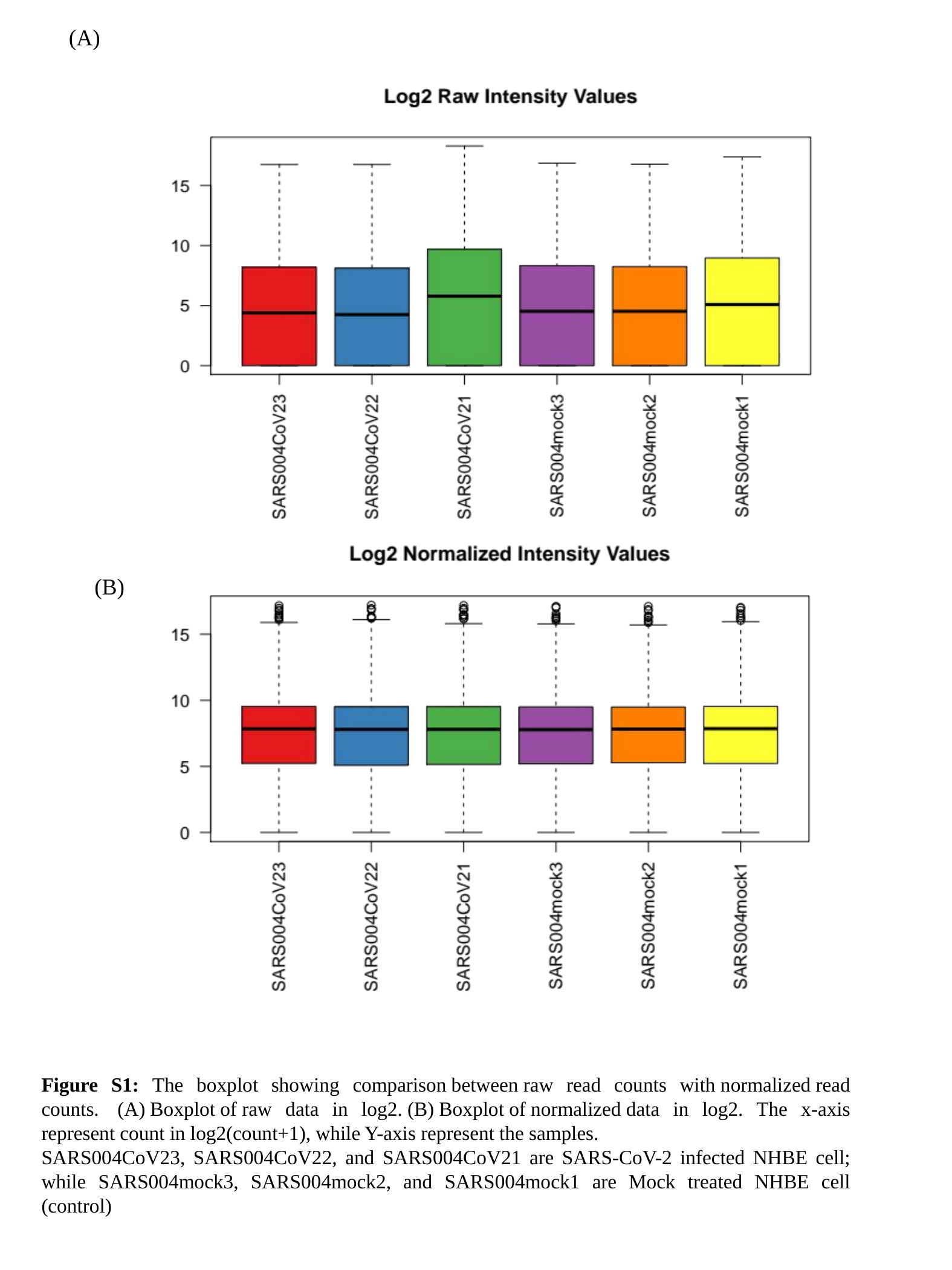

(A)
(B)
Figure S1: The boxplot showing comparison between raw read counts with normalized read counts.  (A) Boxplot of raw data in log2. (B) Boxplot of normalized data in log2. The x-axis represent count in log2(count+1), while Y-axis represent the samples.
SARS004CoV23, SARS004CoV22, and SARS004CoV21 are SARS-CoV-2 infected NHBE cell; while SARS004mock3, SARS004mock2, and SARS004mock1 are Mock treated NHBE cell (control)

## Slide 2
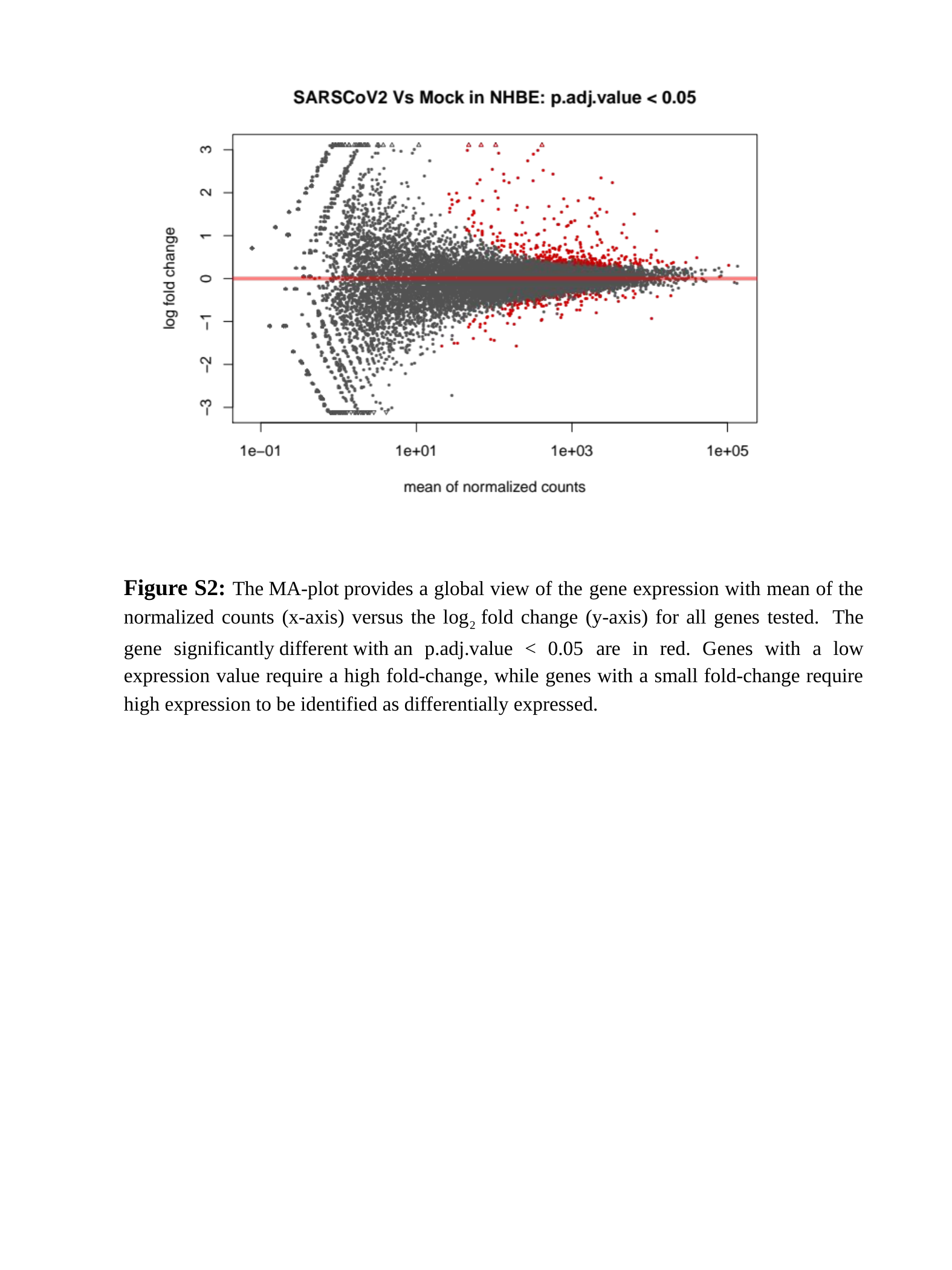

Figure S2: The MA-plot provides a global view of the gene expression with mean of the normalized counts (x-axis) versus the log2 fold change (y-axis) for all genes tested.  The gene significantly different with an p.adj.value < 0.05 are in red. Genes with a low expression value require a high fold-change, while genes with a small fold-change require high expression to be identified as differentially expressed.

## Slide 3
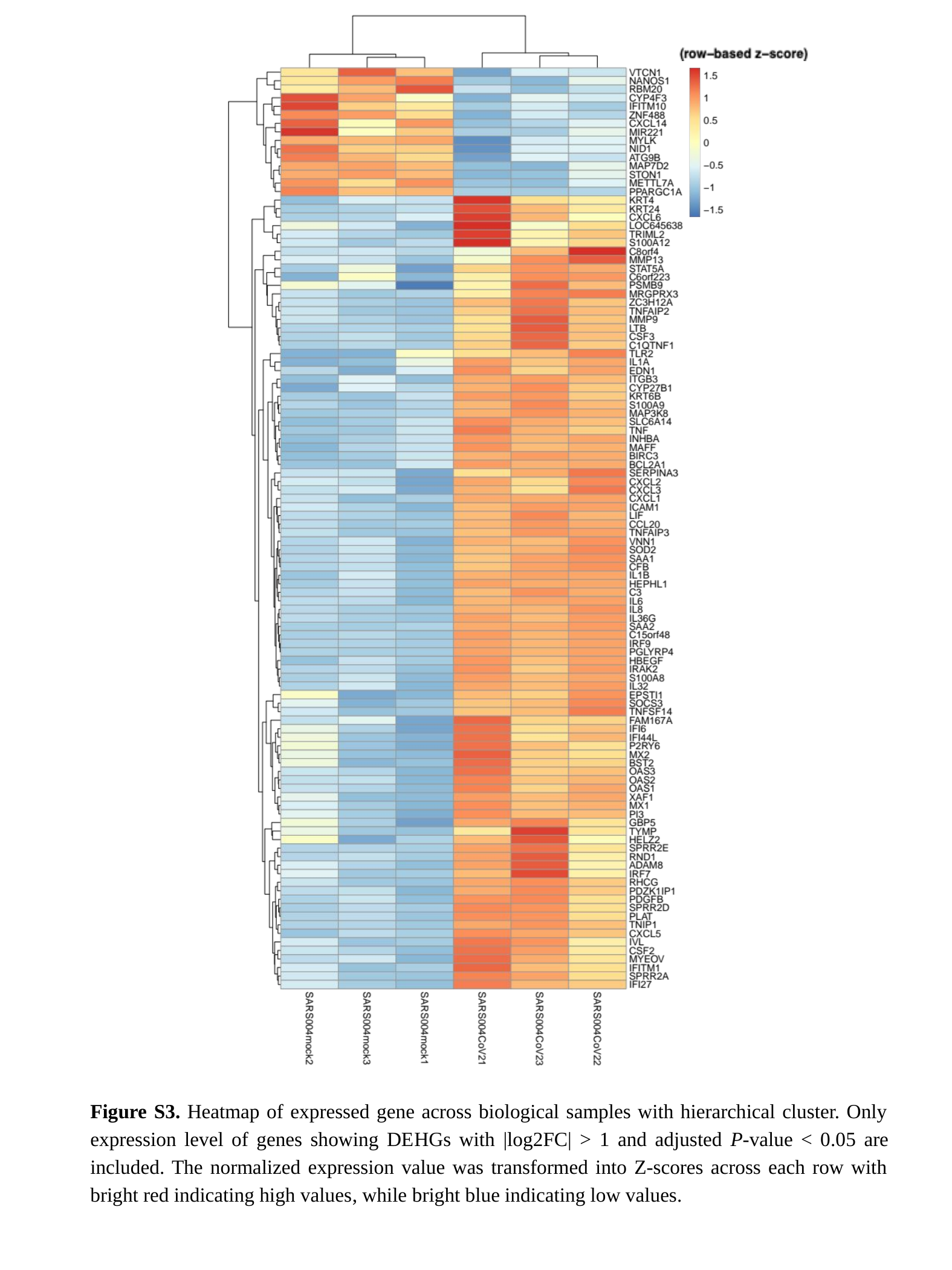

Figure S3. Heatmap of expressed gene across biological samples with hierarchical cluster. Only expression level of genes showing DEHGs with |log2FC| > 1 and adjusted P-value < 0.05 are included. The normalized expression value was transformed into Z-scores across each row with bright red indicating high values, while bright blue indicating low values.

## Slide 4
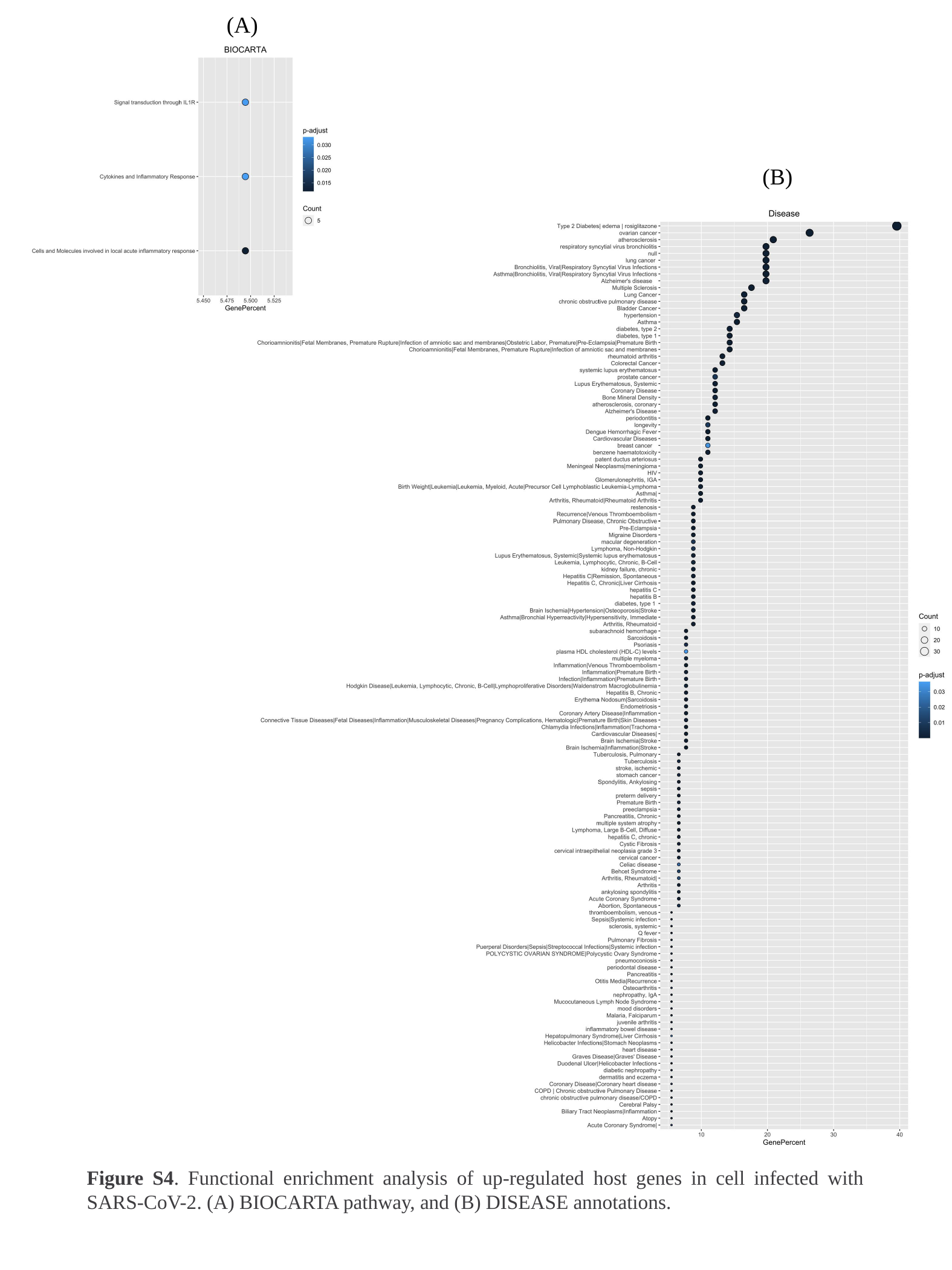

(A)
(B)
Figure S4. Functional enrichment analysis of up-regulated host genes in cell infected with SARS-CoV-2. (A) BIOCARTA pathway, and (B) DISEASE annotations.

## Slide 5
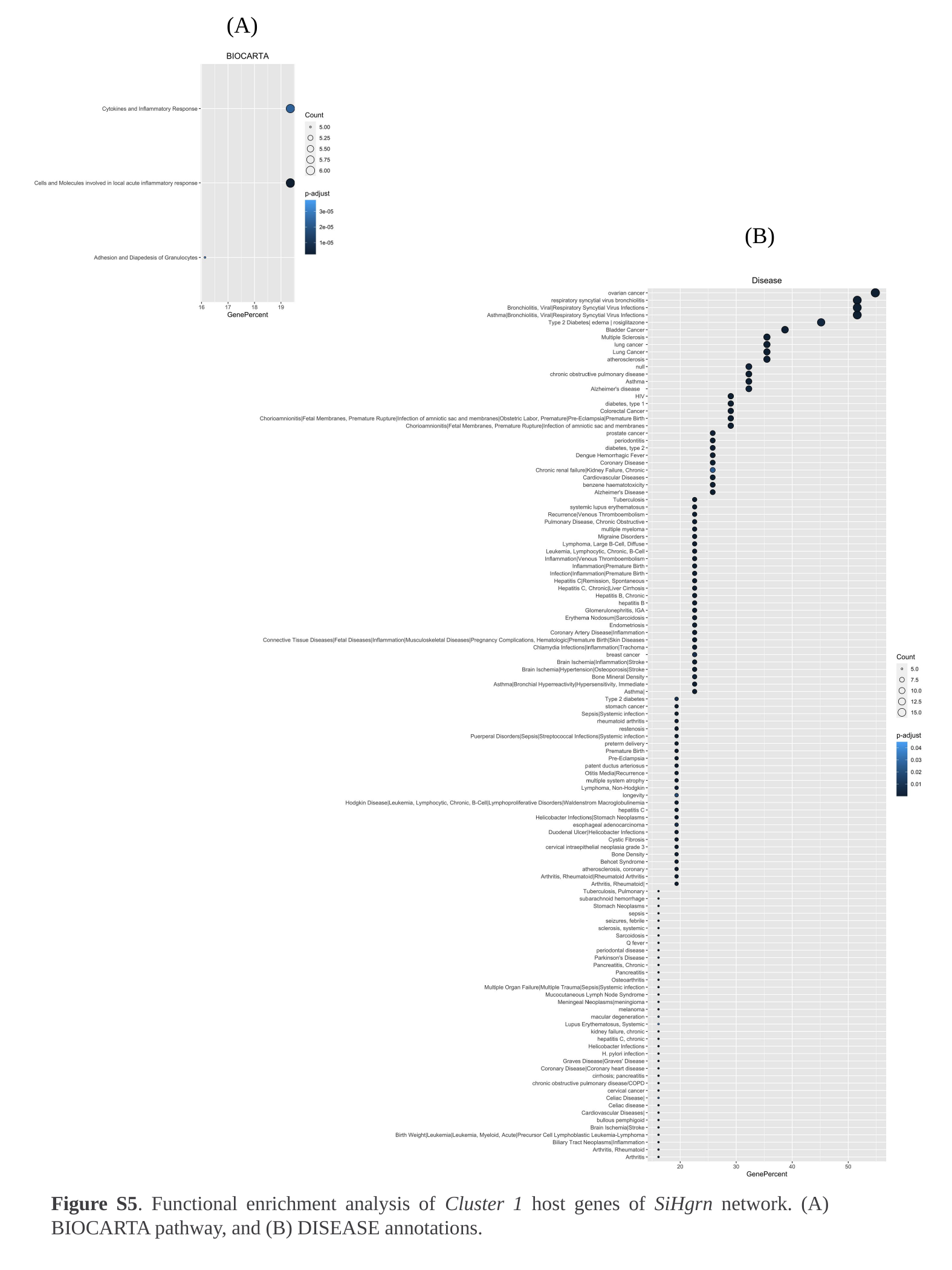

(A)
(B)
Figure S5. Functional enrichment analysis of Cluster 1 host genes of SiHgrn network. (A) BIOCARTA pathway, and (B) DISEASE annotations.

## Slide 6
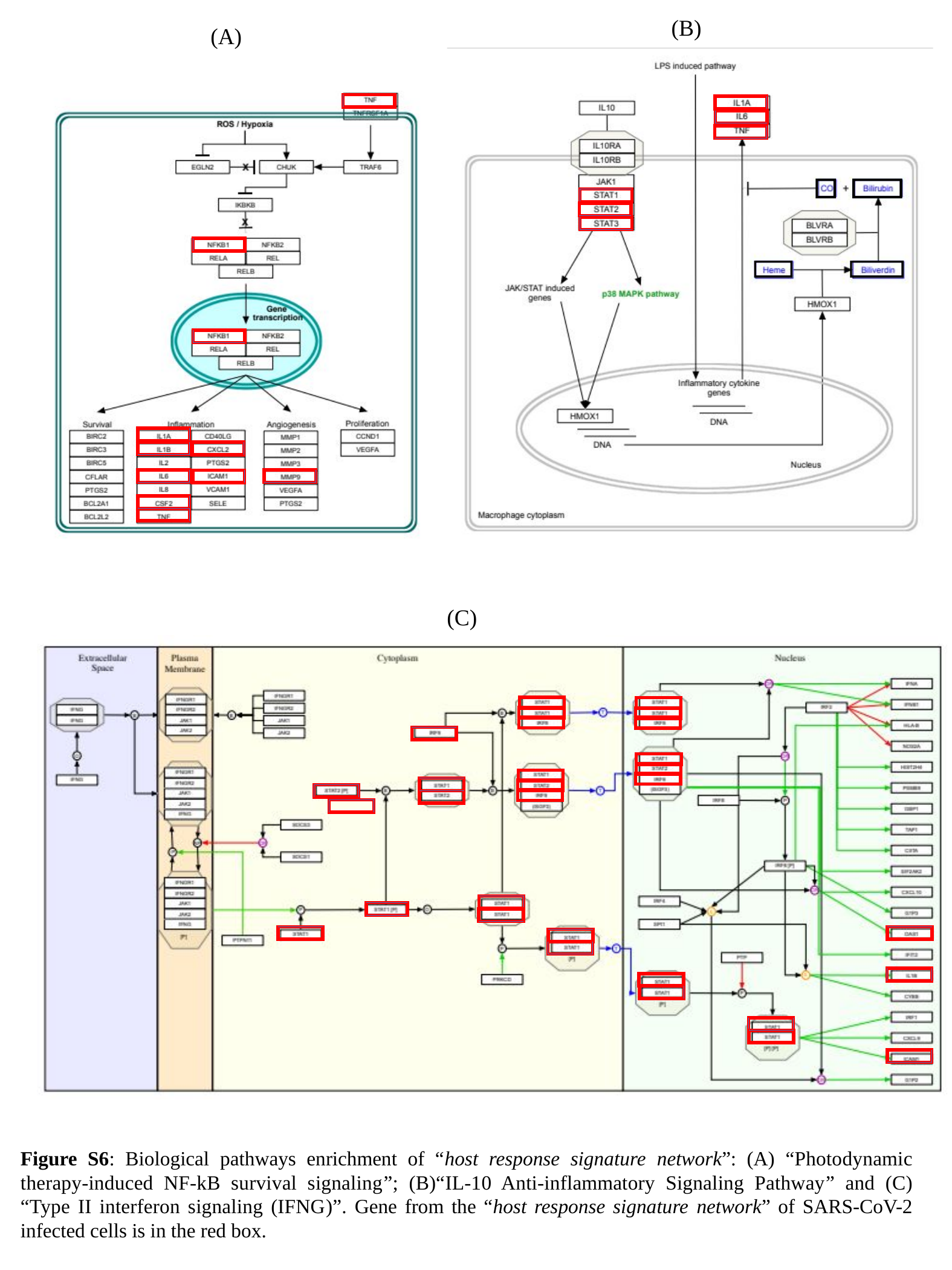

(B)
(A)
(C)
Figure S6: Biological pathways enrichment of “host response signature network”: (A) “Photodynamic therapy-induced NF-kB survival signaling”; (B)“IL-10 Anti-inflammatory Signaling Pathway” and (C) “Type II interferon signaling (IFNG)”. Gene from the “host response signature network” of SARS-CoV-2 infected cells is in the red box.

## Slide 7
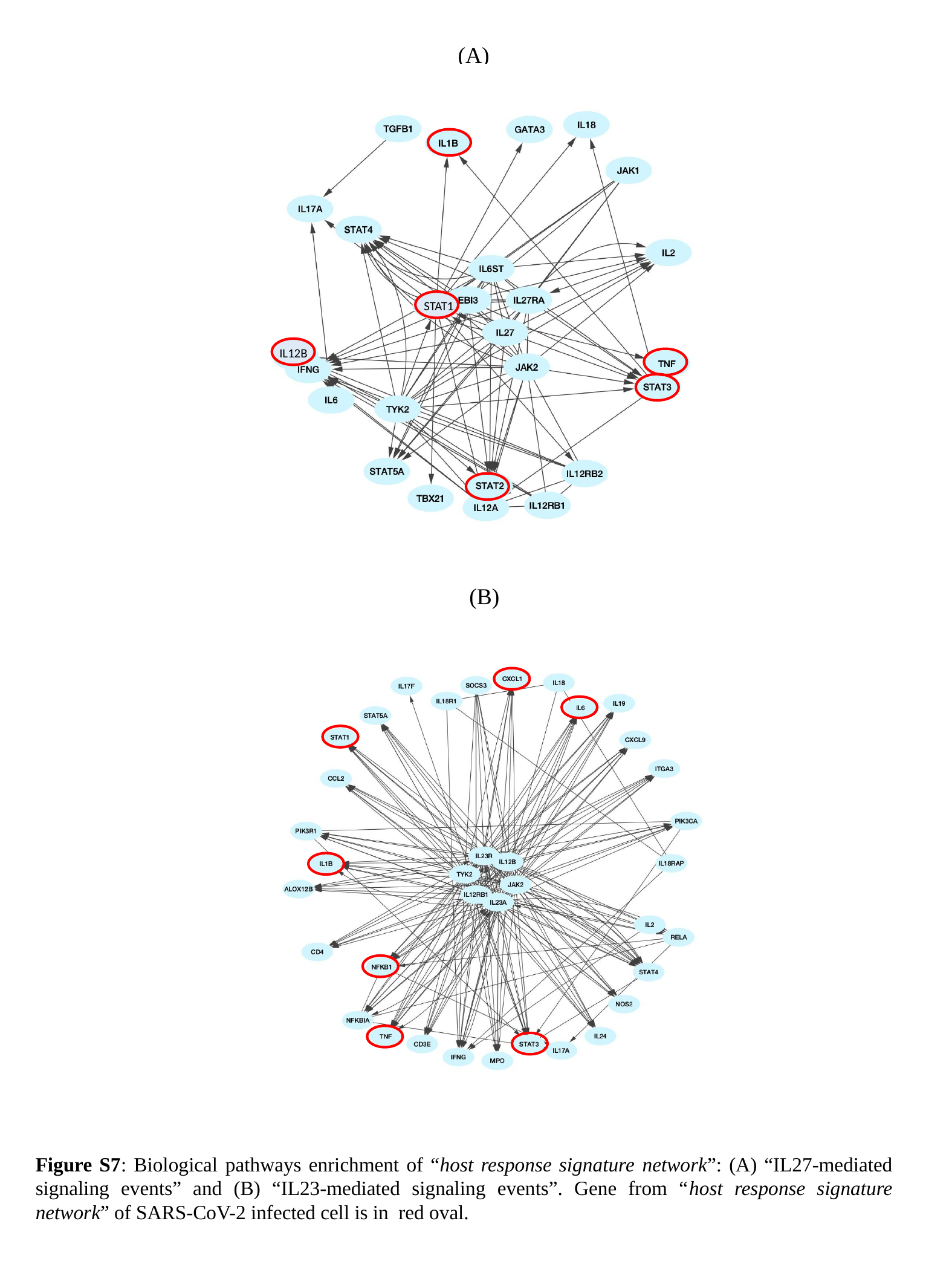

(A)
STAT1
IL12B
(B)
Figure S7: Biological pathways enrichment of “host response signature network”: (A) “IL27-mediated signaling events” and (B) “IL23-mediated signaling events”. Gene from “host response signature network” of SARS-CoV-2 infected cell is in red oval.

## Slide 8
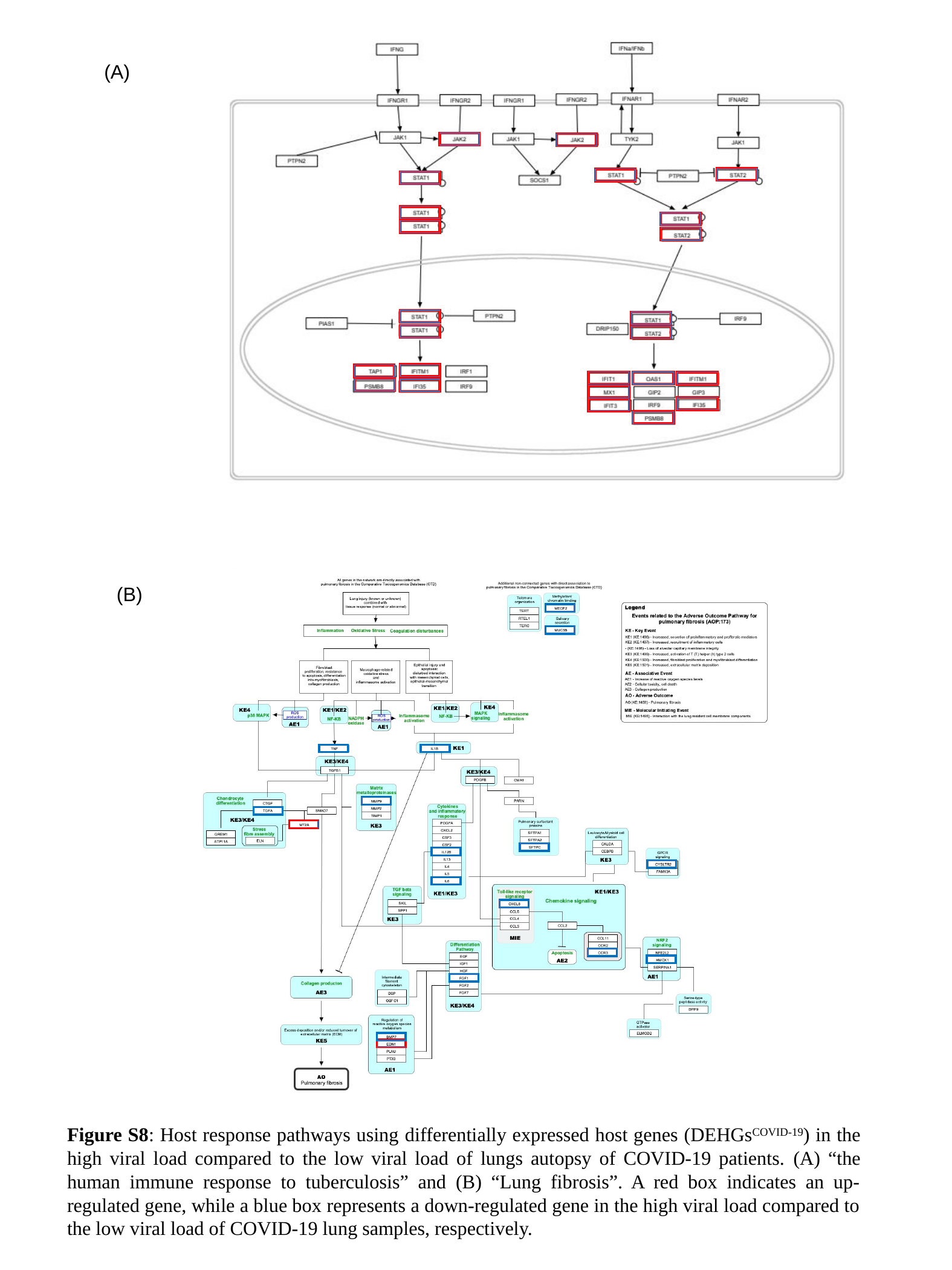

(A)
(B)
Figure S8: Host response pathways using differentially expressed host genes (DEHGsCOVID-19) in the high viral load compared to the low viral load of lungs autopsy of COVID-19 patients. (A) “the human immune response to tuberculosis” and (B) “Lung fibrosis”. A red box indicates an up-regulated gene, while a blue box represents a down-regulated gene in the high viral load compared to the low viral load of COVID-19 lung samples, respectively.

## Slide 9
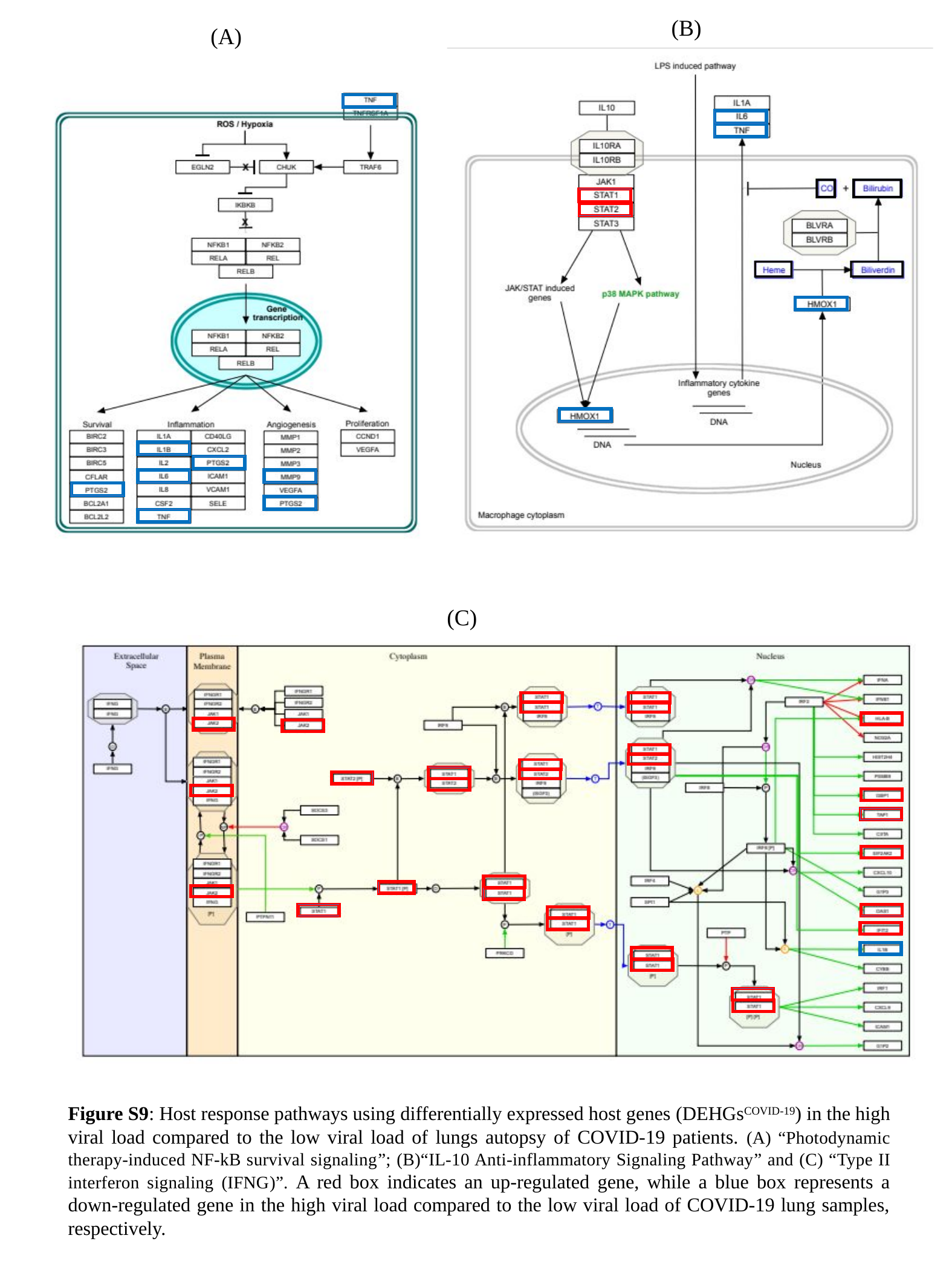

(B)
(A)
(C)
Figure S9: Host response pathways using differentially expressed host genes (DEHGsCOVID-19) in the high viral load compared to the low viral load of lungs autopsy of COVID-19 patients. (A) “Photodynamic therapy-induced NF-kB survival signaling”; (B)“IL-10 Anti-inflammatory Signaling Pathway” and (C) “Type II interferon signaling (IFNG)”. A red box indicates an up-regulated gene, while a blue box represents a down-regulated gene in the high viral load compared to the low viral load of COVID-19 lung samples, respectively.

## Slide 10
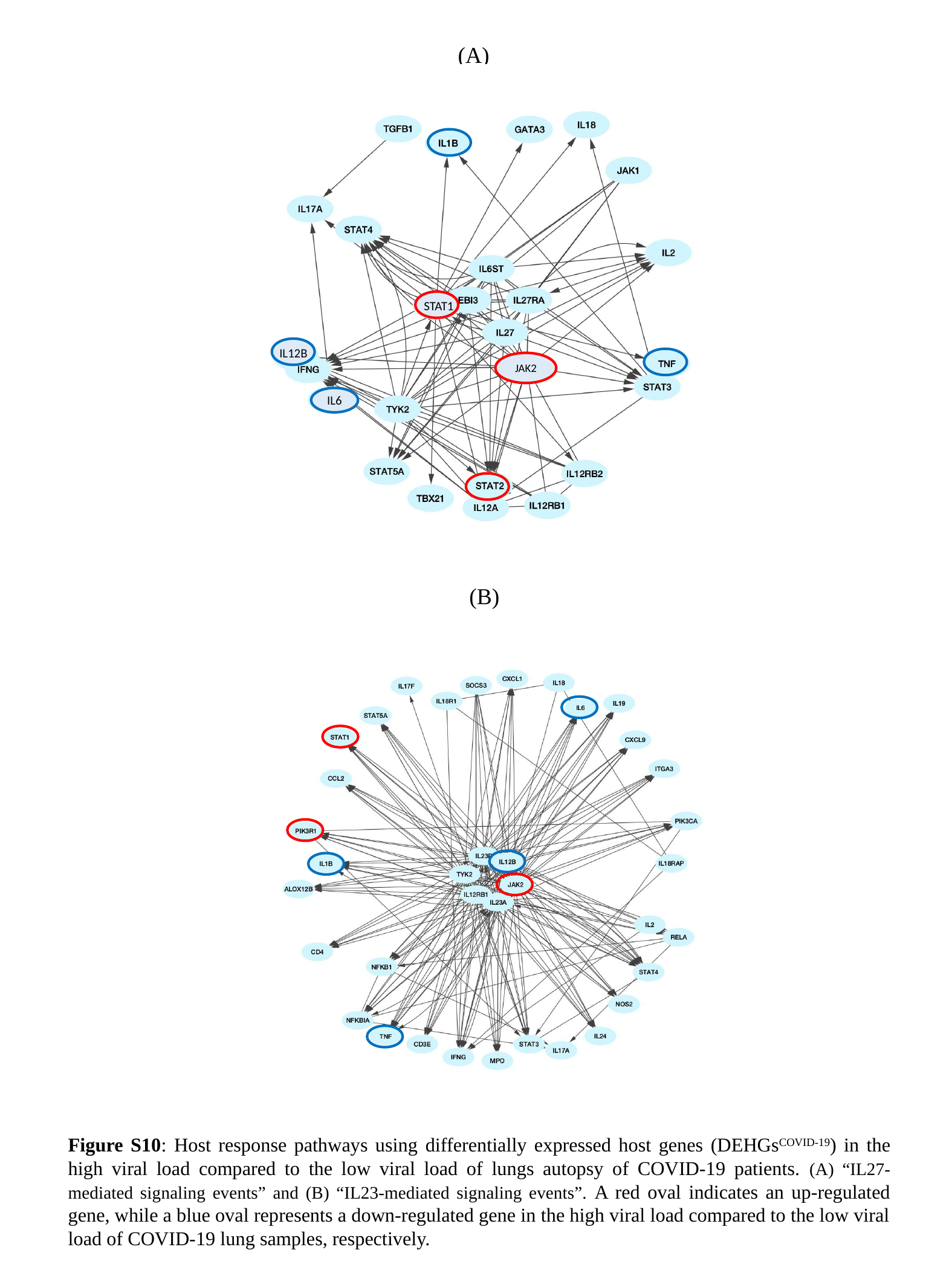

(A)
STAT1
IL12B
JAK2
IL6
(B)
Figure S10: Host response pathways using differentially expressed host genes (DEHGsCOVID-19) in the high viral load compared to the low viral load of lungs autopsy of COVID-19 patients. (A) “IL27-mediated signaling events” and (B) “IL23-mediated signaling events”. A red oval indicates an up-regulated gene, while a blue oval represents a down-regulated gene in the high viral load compared to the low viral load of COVID-19 lung samples, respectively.
